# Genome-Wide Identification of Long Non-Coding RNAs and Their Regulatory Networks Involved in *Apis mellifera ligustica* Response to *Nosema ceranae* Infection

**DOI:** 10.3390/insects10080245

**Published:** 2019-08-09

**Authors:** Dafu Chen, Huazhi Chen, Yu Du, Dingding Zhou, Sihai Geng, Haipeng Wang, Jieqi Wan, Cuiling Xiong, Yanzhen Zheng, Rui Guo

**Affiliations:** College of Bee Science, Fujian Agriculture and Forestry University, Fuzhou 350002, China

**Keywords:** long non-coding RNA, competitive endogenous RNA, regulatory network, honeybee, *Nosema ceranae*, stress response, immune defense

## Abstract

Long non-coding RNAs (lncRNAs) are a diverse class of transcripts that structurally resemble mRNAs but do not encode proteins, and lncRNAs have been proven to play pivotal roles in a wide range of biological processes in animals and plants. However, knowledge of expression patterns and potential roles of honeybee lncRNA response to *Nosema ceranae* infection is completely unknown. Here, we performed whole transcriptome strand-specific RNA sequencing of normal midguts of *Apis mellifera ligustica* workers (Am7CK, Am10CK) and *N. ceranae*-inoculated midguts (Am7T, Am10T), followed by comprehensive analyses using bioinformatic and molecular approaches. A total of 6353 *A. m. ligustica* lncRNAs were identified, including 4749 conserved lncRNAs and 1604 novel lncRNAs. These lncRNAs had minimal sequence similarities with other known lncRNAs in other species; however, their structural features were similar to counterparts in mammals and plants, including shorter exon and intron length, lower exon number, and lower expression level, compared with protein-coding transcripts. Further, 111 and 146 *N. ceranae*-responsive lncRNAs were identified from midguts at 7-days post-inoculation (dpi) and 10 dpi compared with control midguts. Twelve differentially expressed lncRNAs (DElncRNAs) were shared by Am7CK vs. Am7T and Am10CK vs. Am10T comparison groups, while the numbers of unique DElncRNAs were 99 and 134, respectively. Functional annotation and pathway analysis showed that the DElncRNAs may regulate the expression of neighboring genes by acting in *cis* and *trans* fashion. Moreover, we discovered 27 lncRNAs harboring eight known miRNA precursors and 513 lncRNAs harboring 2257 novel miRNA precursors. Additionally, hundreds of DElncRNAs and their target miRNAs were found to form complex competitive endogenous RNA (ceRNA) networks, suggesting that these DElncRNAs may act as miRNA sponges. Furthermore, DElncRNA-miRNA-mRNA networks were constructed and investigated, the results demonstrated that a portion of the DElncRNAs were likely to participate in regulating the host material and energy metabolism as well as cellular and humoral immune host responses to *N. ceranae* invasion. Our findings revealed here offer not only a rich genetic resource for further investigation of the functional roles of lncRNAs involved in the *A. m. ligustica* response to *N. ceranae* infection, but also a novel insight into understanding the host-pathogen interaction during honeybee microsporidiosis.

## 1. Introduction

Honeybees are pivotal pollinators of crops and wild flora, and of great importance in supporting critical ecosystem balance [1]. In addition, honeybees serve as key models for studies on development, social behavior, disease transmission, and host-pathogen interaction [2]. The western honeybee (*Apis mellifera*) has been domesticated for honey production and crop pollination all over the world. The genome of *A. mellifera* was published in 2006 [3], which laid a solid foundation for its molecular and functional genomics studies. *Apis mellifera ligustica*, a subspecies of *A. mellifera*, is widely used in the beekeeping industry in China and many other countries.

Microsporidia are spore-forming and obligate intracellular fungal pathogens that can infect a wide variety of hosts such as mammals and insects [4]. Microsporidia infection occurs through the ingestion of spores from contaminated food or water, followed by the germination of these spores that are activated by the physical and chemical conditions inside the midgut; subsequently, the sporoplasm is injected into the host midgut epithelium, where it multiplies; ultimately, the spores are excreted from the host in the feces, offering new sources of infection through cleaning and feeding activities inside the colonies, or they are disseminated into the environment [5,6,7]. Nosemosis is a serious disease in adult honeybees resulting from infection with *Nosema* species including *Nosema apis* and *Nosema ceranae*. The latter is a widespread microsporidian pathogen of honeybees, which was first identified by Fries et al. from *A. cerana* near Beijing [8], China; shortly afterward, it was reported to have spread to Europe [9] and Taiwan [10]. Currently, *N. ceranae* has been found in colonies of western honeybee throughout the world [11,12]. *N. ceranae* is infective to all castes in the colony [7], and it could dramatically reduce colony strength and productivity [13] as well as interacts with other environmental stressors to severely weaken colony health [14,15].

Previous studies revealed that much of the genome is transcribed, but only a small fraction of sequences possess protein-coding capacity [16]. In humans, less than 2% of the genome contains conserved sequences for proteins [16]. Hence, many of the transcribed sequences in the genome are believed to be non-coding RNAs (ncRNAs), which are arbitrarily categorized into two types according to their sizes; one is small RNAs shorter than 200 nt, such as microRNAs (miRNAs) and small nucleolar RNAs (snoRNAs); the other type is long non-coding RNAs (lncRNAs), which are longer than 200 nt and lack protein-coding potential [17]. LncRNAs can be further classified into antisense lncRNAs, intronic lncRNAs, overlapping lncRNAs, and intergenic lncRNAs [18]. LncRNAs are usually expressed at low levels, lack conservation among species, and often display tissue- or cell-specific expression patterns [19,20]. In recent years, lncRNAs have been found to play key roles in various biological processes in mammals and plants as potent regulators, natural miRNA target mimics, chromatin modifiers, and molecular cargo for protein relocalization [21]. Additionally, lncRNAs have been found to be closely related to some diseases such as Alzheimer’s disease [22] and acquired immune deficiency syndrome (AIDS) [23], and therefore can be used as novel biomarkers and therapeutic targets. With the rapid development of high-throughput sequencing techniques, genome-wide investigations for lncRNAs have been conducted via cDNA/EST *in silico* mining [24,25], whole-genome tiling array [26], and RNA-seq approaches [27]. By using deep sequencing and bioinformatics, more than 8000 lncRNAs have been predicted in humans [19] and approximately 4000 lncRNAs have been identified in mice [28,29]. In plants, 6480 transcripts have been classified as lncRNAs in *Arabidopsis* [30]; 125 putative stress-responsive lncRNAs have been identified in wheat [31]. In microorganisms, our research group identified 379 novel lncRNAs in *Ascosphaera apis* (another common fungal pathogen of honeybee), along with 83 in *N. ceranae*, and revealed that these fungal lncRNAs share similar characteristics with those in mammals and plants, such as shorter length and reduced exon number [32,33]. Recently, lncRNAs were identified in insects such as *Plutella xylostella* [34], *Anopheles gambiae* [35], and *Bombycis mori* [36]. However, compared with mammals and plants, knowledge of honeybee lncRNAs remains largely unknown. Thus far, only few lncRNAs have been discovered in honeybee, such as *lncov1*, *lncov2*, and *Ks-1* [37,38]. Utilizing transcriptome sequencing, Jayakodi et al. [39] identified 1514 long intergenic non-coding RNAs (lincRNAs) in *A. mellifera* and 2470 lincRNAs in *Apis cerana*, most of which had a tissue-specific expression pattern. More recently, Chen et al. [40] predicted a variety of lncRNAs, miRNAs, and mRNAs during ovary activation, oviposition inhibition and oviposition recovery processes; they further found 73 differentially expressed genes (DEGs) and 14 differentially expressed lncRNAs (DElncRNAs) located in the QTL region, which may be candidate genes responsible for ovary size and oviposition.

To our knowledge, no study on honeybee lncRNA response to fungal stress was reported until now, and understanding of the potential roles of host stress-responsive lncRNAs were extremely limited. Here, to systematically identify lncRNAs, corresponding regulatory networks, and their potential roles involved in the *A. m. ligustica* response to *N. ceranae* stress, we first performed whole transcriptome strand-specific RNA sequencing of normal and *N. ceranae*-infected midgut samples of *A. m. ligustica* workers. We examined the expression patterns of host lncRNAs responding to *N. ceranae* challenge, followed by molecular validation of differentially expressed DElncRNAs. Moreover, regulatory networks of *A. m. ligustica* DElncRNAs were constructed and analyzed to further explore their potential roles during the fungal stress response. The current work generated a comprehensive list of *A. m. ligustica* lncRNAs, which will be a valuable complement to the other ncRNAs that have already been discovered in this important social insect. The results not only lay a foundation for deciphering the molecular mechanisms underlying the *A. m. ligustica* response to *N. ceranae* stress, but also offer a beneficial resource for functional study of key *N. ceranae*-responsive lncRNAs in the future. Our data can also help generate better understanding of honeybee-microsporidia interactions.

## 2. Materials and Methods

### 2.1. N. ceranae Spore Purification

Fresh spores were isolated from naturally infected foragers from a colony located in Fuzhou city, Fujian province, China, following the method described by Cornman et al. [41] with some modifications [33]. (1) Bees were kept at −20 °C for 5 min to anesthetize them, followed by separation of midguts with clean dissection tweezers, homogenization in distilled water, filtration through four layers of sterile gauze, and then three cylces of centrifugation at 6000× *g* for 5 min; (2) the supernatant was discarded as the spores remained in the sediment, and the resuspended pellet was further purified on a discontinuous Percoll gradient (Solarbio) consisting of 5 mL each of 25%, 50%, 75% and 100% Percoll solution; the spore suspension was then overlaid onto the gradient and centrifuged at 18,000× *g* for 90 min at 4 °C; (3) the spore pellet was carefully extracted with a sterile syringe and then centrifuged again on a discontinuous Percoll gradient to obtain clean spores (Figure S1A), which were frozen in liquid nitrogen and stored at −80 °C until deep sequencing, RT-PCR, and real-time quantitative PCR (RT-qPCR). An aliquot of spores was subjected to PCR identification and confirmed to be mono-specific using previously described primers [12]. The spore concentration was determined by counting using a CL kurt counter (JIMBIO) and the suspension was freshly prepared before use.

### 2.2. Experimental Design and Sample Collection

A *N. ceranae*-free colony of *A. m. ligustica* located in the teaching apiary of the College of Bee Science in Fujian Agriculture and Forestry University was selected for experimental work. No *Varroa* was observed before and during the whole experiment. RT-PCR was used to detect the prevalence of several common bee viruses (DWV, KBV, IAPV, CBPV, BQCV, ABPV, and SBV) and *N. ceranae* in the emergent honeybee with previously developed specific primers (Table S1) [42,43,44,45,46,47]. As shown in Figure S2, only DWV was detected. Frames of a sealed brood were kept in an incubator at 34 ± 0.5 °C with 50% RH to provide newly emerged *Nosema*-free honeybees. The emergent workers were carefully removed, confined to cages in groups of 20, and kept in the incubator at 32 ± 0.5 °C with 50% RH. The bees were fed *ad libitum* with a solution of sucrose (50% w/w in water). One day after eclosion, the honeybees were starved for 2 h and 20 workers per group were each immobilized and then fed with 5 μL of 50% sucrose solution containing 1 × 10^6^ spores of *N. ceranae* (Figure S1B). There are two main reasons responsible for this inoculation dose. Firstly, the dose was also detected in naturally infected bees [48]; secondly, 1.25 × 10^5^ [15] and 1 × 10^4^ *N. ceranae* spores [49] were previously used to inoculate bees in other studies, thus 1 × 10^6^ spores were used for inoculation of workers in this work to guarantee an effective infection. Those individuals that did not consume the total amount of solution were discarded from the assay. After feeding, bees were isolated for 30 min in individual vials in the growth chamber to ensure that the sugar solution was not transferred among honeybees and that the entire dosage was ingested. Control bees were inoculated in an identical manner using a 50% sucrose solution (w/w in water) without *N. ceranae* spores. Three replicate cages of 20 honeybees each were used in *N. ceranae*-treated and control groups. Each cage was checked every 24 h, and any dead bees were removed. *N. ceranae*-treated and control workers’ midguts were respectively harvested 7 d or 10 d post-inoculation (dpi), immediately frozen in liquid nitrogen and kept at −80 °C until high-throughput sequencing and molecular experiment. Treatment groups corresponding to 7 dpi and 10 dpi with sucrose solution containing *N. ceranae* spores were termed as Am7T (Am7T-1, Am7T-2, and Am7T-3 were three biological replicas) and Am10T (Am10T-1, Am10T-2, and Am10T-3 were three biological replicas); control groups 7 dpi and 10 dpi with sucrose solution without *N. ceranae* spores were termed as Am7CK (Am7CK-1, Am7CK-2, and Am7CK-3 were three biological replicas) and Am10CK (Am10CK-1, Am10CK-2, and Am10CK-3 were three biological replicas). Each replica contains three midguts. Here, the timeline (7 d and 10 d) used in this study was on basis of two main points. Firstly, a life circle of *N. ceranae* is 6 d [50]; secondly, the number of *N. ceranae* is increasing in infected western honeybee during a period of 10–20 d [51].

### 2.3. Mortality Rate Analysis

In parallel, the inoculation experiment was conducted using the aforementioned method. The number of dead bees was recorded each day until 10 dpi. The accumulated mortality rate was calculated and analyzed with GraphPad prism 7.0 (GraphPad).

### 2.4. RNA Extraction, Strand-Specific cDNA Library Construction and Deep Sequencing

Total RNA of the six biological replicas (Am7T-1, Am7T-2, Am7T-3, Am10T-1, Am10T-2, and Am10T-3) from *N. ceranae*-treated groups and six biological replicas (Am7CK-1, Am7CK-2, Am7CK-3, Am10CK-1, Am10CK -2, and Am10CK-3) from control groups were respectively extracted using Trizol (Life Technologies) following the manufacturer’s instructions, and checked via 1% agarose gel eletrophoresis. Subsequently, rRNAs were removed to retain mRNAs and ncRNAs, which were fragmented into short fragments by using fragmentation buffer (Illumina) and reverse transcripted into cDNA with random primers. Next, second-strand cDNA were synthesized by DNA polymerase I, RNase H, dNTP (dUTP instead of dTTP), and buffer. The cDNA fragments were purified using QiaQuick PCR extraction kit (QIAGEN), end repaired, poly (A) added, and ligated to Illumina sequencing adapters. UNG (Uracil-N-Glycosylase) (Illumina) was then used to digest the second-strand cDNA. Ultimately, the digested products were size selected by agarose gel electrophoresis, PCR amplified, and sequenced on Illumina HiSeq^TM^ 4000 platform (Illumina) by Gene Denovo Biotechnology Co. (Guangzhou). All RNA sequencing data produced in our study are available in NCBI Short Read Archive database (http://www.ncbi.nlm.nih.gov/sra/) and could be available on Sequence Read Archive (SRA) database and connected to BioProject PRJNA406998.

### 2.5. Quality Control and Mapping of Reads

Reads produced from the sequencing machines included raw reads containing adapters or low-quality bases which would affect the following assembly and analysis. Therefore, reads were further filtered by removing reads containing adapters, more than 10% of unknown nucleotides (N), and more than 50% of low quality (*Q*-value ≤ 20) bases to gain high quality clean reads.

Short reads alignment tool Bowtie2 [52] was used for mapping reads to ribosome RNA (rRNA) database. The mapped reads were then removed and the remaining reads were further used in assembly and analysis of transcriptome. The rRNA removed reads of each sample were then mapped to reference genome of *Apis mellifera* (assembly Amel_4.5) by TopHat2 (version 2.0.3.12) [53]. The alignment parameters were as follows: (1) maximum read mismatch is two; (2) the distance between mate-pair reads is 50 bp; (3) the error of distance between mate-pair reads is ±80 bp.

### 2.6. Transcripts Assembly

Transcripts were assembled using software Cufflinks [54], which together with TopHat2, allow researchers to identify novel genes and novel splice variants of known ones. The program reference annotation-based transcripts (RABT) was preferred. Cufflinks constructed faux reads according to reference to make up for the influence of low coverage sequencing. During the last step of assembly, all of the reassembles fragments were aligned with reference genes and then similar fragments were removed. Cuffmerge was used to merge transcripts from different replicas of a group into a comprehensive set of transcripts, and the transcripts from multiple groups were then merged into a finally comprehensive set of transcripts for further downstream analyses.

### 2.7. Bioinformatic Pipeline for Identification and Annotation of lncRNAs, and Quantification

To identify the novel transcripts, all of the reconstructed transcripts were aligned to reference genome of *Apis mellifera* (assembly Amel_4.5) and were divided into twelve categories by using Cuffcompare [48]. Transcripts with one of the classcodes “u, i, j, x, c, e, o” were defined as novel transcripts. The following parameters were used to identify reliable novel lncRNAs: the length of transcript was longer than 200 bp and the exon number was more than two; novel transcripts were then aligned to the Nr, GO (Gene Ontology) and KEGG (Kyoto Encyclopedia of Genes and Genomes) databases to obtain protein functional annotation. Softwares CNCI [55] and CPC [56] were utilized in combination to sort non-protein-coding RNA candidates from putative protein-coding RNAs in the unknown transcripts by default parameters. The intersection of both results was chosen as lncRNAs. The different types of lncRNAs including lincRNA, intronic lncRNA, anti-sense lncRNA were selected using cuffcompare. The detailed flow of novel lncRNA prediction is shown in Figure S3.

Transcripts abundances were quantified by software RSEM [57] following (1) a set of reference transcript sequences were generated, preprocessed according to known transcripts, new transcripts (in FASTA format), and gene annotation files (in GTF format); (2) reads were realigned to the reference transcripts by Bowtie alignment program and the resulting alignments were used to estimate transcript abundances.

The transcript expression level was normalized by using FPKM (fragments per kilobase of transcript per million mapped reads) method, which can eliminate the influence of different transcripts lengths and sequencing data amount on the calculation of transcripts expression. Therefore, the calculated transcripts expression can be directly used for comparing the difference of transcripts expression among samples.

### 2.8. DElncRNAs, Target Gene, and ceRNA Analyses

DElncRNAs between any two libraries were identified by edgeR [58] (release 3.2). A statistical analysis of the frequency of all transcripts and their corresponding *p*-values were performed with the method described by Audic et al. [59]. The significance threshold of *p*-value in multiple tests was set by false discovery rate (FDR). The thresholds used to evaluate the statistical significance of differences in lncRNA expression were defined as FDR < 0.05 and an absolute value of the log2 (Fold change) > 1.

*Cis*-acting lncRNAs function via targeting neighbouring genes [60,61]. In the present study, we searched for coding genes in the regions located 10-kb upstream and downstream of all of the identified lncRNAs for predicting their functional roles. *Trans*-acting lncRNAs function via interaction with co-expressed genes. We searched for co-expressed coding genes of all lncRNAs and reserved the most positively and negatively correlated ones for predicting their functional roles.

All neighbouring genes and co-expressed genes were mapped to GO terms in the GO database (http://www.geneontology.org/), and gene numbers were calculated for each term; significantly enriched GO terms in neighbouring genes and co-expressed genes comparing to the reference genome background were defined by hypergeometric test. KEGG pathway enrichment analysis was conducted using KOBAS 2.0, with the *A. mellifera* genome as background. Only GO terms or KEGG pathways with corrected *p*-values of less than 0.05 were considered enriched.

Following traditional miRNA target prediction methods, we inferred the conserved regions of *A. m. ligustica* lncRNAs that may harbor MREs for ceRNA networks. miRanda(v3.3a) [62] (animal), RNAhybrid(v2.1.2)+svm_light(v6.01) [63,64] (animal) and TargetFinder(Version: 7.0) [65] (plant) were used to predict MREs in the conserved regions of lncRNAs.

### 2.9. Real-time quantitative PCR (RT-qPCR) confirmation of DElncRNAs

To validate our RNA-seq data, seven DElncRNAs were randomly selected for RT-qPCR assays, including three (TCONS_00003147, TCONS_00008930, and TCONS_00003072) from Am7CK vs. Am7T and four (XR_001705522.1, TCONS_00032699, XR_001705654.1, and TCONS_00012311) from Am10CK vs. Am10T. The first cDNA strand was synthesized using the SuperScript first-strand synthesis system (Yeasen) according to the protocol. Primers for qPCR were designed using the DNAMAN software and synthesized by Sangon Biotech Co., Ltd. The housekeeping gene *actin* was used as an internal control. The RNA samples used as templates for RNA-seq were the same as those used for RT-qPCR, which was conducted on a QuanStudio Real-Time PCR System (ThemoFisher). The 20 μL PCR reaction mixture contained 10 μL SYBR Green dye (Yeasen); 0.4 μL (10 pmol/μL) specific forward primer; 0.4 μL (10 pmol/μL) reverse primer; 0.4 μL ROX reference dye; 2 μL (10 ng/μL) diluted cDNA; and 6.8 μL RNase free water. Cycling parameters were as follows: 95 °C for 1 min, followed by 40 cycles of 95 °C for 15 s, 60 °C for 30 s, and 72 °C for 45 s. The relative gene expression was calculated using the 2^–ΔΔCT^ method [66]. These assays were performed in triplicate. The specific primers used in RT-qPCR are shown in Table S2.

### 2.10. Statistical Analysis

All statistical analyses were performed using SPSS software (IBM) and GraphPad Prism 7.0 software (GraphPad). Data were presented as mean ± standard deviation (SD). Statistics analysis was conducted using independent-samples t-test, Log-rank test. Fisher’s exact test was employed to filter the significant GO terms and KEGG pathways using R software 3.3.1. *p* < 0.05 was considered statistically significant.

## 3. Results

### 3.1. The Effect of N. ceranae Inoculation Dose on the Mortality of A. m. ligustica Workers

**Figure 1:**
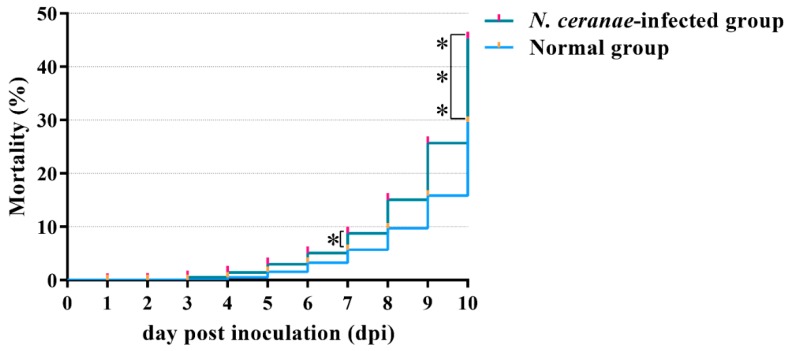
The accumulated mortality rate of *A. m. ligustica* workers in *N. ceranae*-infected group and normal group. Log-rank test: * indicates *p* < 0.05; *** indicates *p* < 0.0001.

Here, each worker (1 d) in treatment group was inoculated with 1 × 10^6^ *N. ceranae* spores. To confirm the effectiveness of this dose, the accumulated mortality rate of workers in *N. ceranae*-infected group and control group was analyzed. As Figure 1 shown, the cumulated mortality rate of workers in both groups increased over time, but the cumulated mortality rate of workers in *N. ceranae*-infected group was significantly higher than that of workers in control group at both 7 dpi (Log-rank test: *p* < 0.05) and 10 dpi (Log-rank test: *p* < 0.0001). This is similar to previous findings [67,68]. Hence, the workers’ midguts at 7 dpi and 10 dpi were used for deep sequencing.

### 3.2. Sequencing Results and Quality Control

In our study, a total of 1,956,129,858 raw reads were produced from 12 cDNA libraries, and 1,946,489,304 clean reads were obtained after strict quality control (Table 1). The percentage of clean reads among raw reads in each library ranged from 99.42% to 99.57%, with a mean Q30 of 93.82% (Table 1). In addition, clean reads were aligned with the reference genome of *A. mellifera*, and the result showed that mapping ratios of 12 samples ranged from 39.48% to 60.76%. Among these mapped reads, 66.17–70.07% were mapped to coding DNA sequence regions, 5.34–8.18% to intron regions, 14.49–16.08% to intergenic regions, and 8.83–11.00% to untranslated regions. Moreover, high Pearson correlation coefficients (0.9119–0.9993) were found among biological replicas within each group, suggesting the reproducibility of sample preparation (Figure S4).

### 3.3. Characterization and Validation of A. m. ligustica lncRNAs

**Figure 2:**
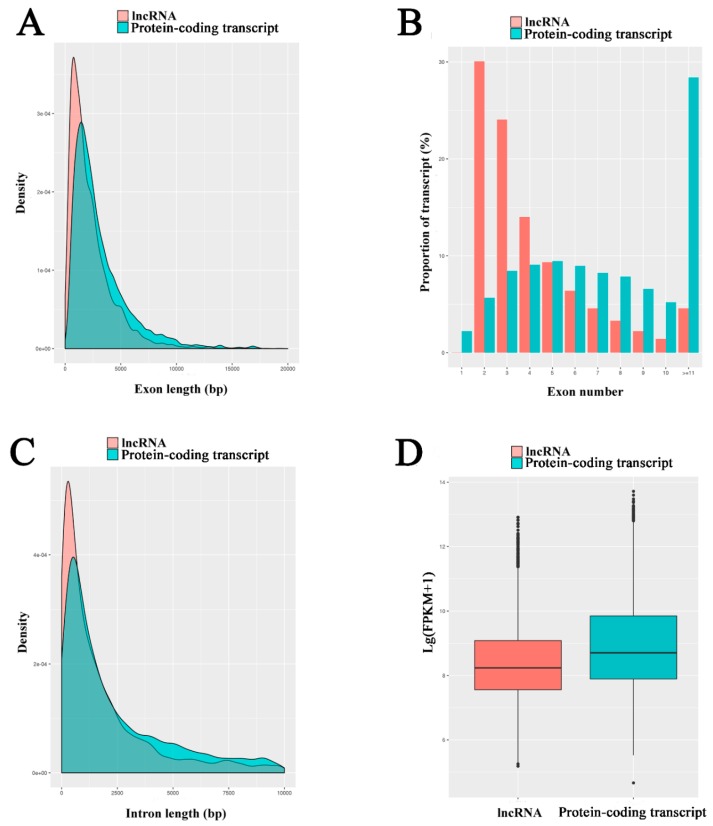
Properties of *A. m. ligustica* long non-coding RNAs (lncRNAs). (**A**) exon size distributions for lncRNAs and protein-coding transcripts. (**B**) number of exons per lncRNAs and protein-coding transcripts. (**C**) intron size distributions for lncRNAs and protein-coding transcripts. (**D**) expression levels of lncRNAs and protein-coding transcripts.

A high stringency filtering process (presented in Figure S3) was used to remove low quality lncRNA transcripts. In total, 6353 lncRNAs were identified from midgut samples, including 4749 known lncRNAs and 1604 novel lncRNAs. These *A. m. ligustica* lncRNAs were found to be shorter in exon and intron length and fewer in exon number than protein-coding genes (Figure 2A–C), which is in accordance with findings in previous studies [69,70,71,72,73]. Additionally, the expression level of each transcript was estimated, and the result indicated that the levels of lncRNAs were lower than those of mRNAs in the midgut of *A. m. ligustica* worker (Figure 2D).

### 3.4. Identification of A. m. ligustica lncRNAs that Respond to N. ceranae Stress

**Figure 3:**
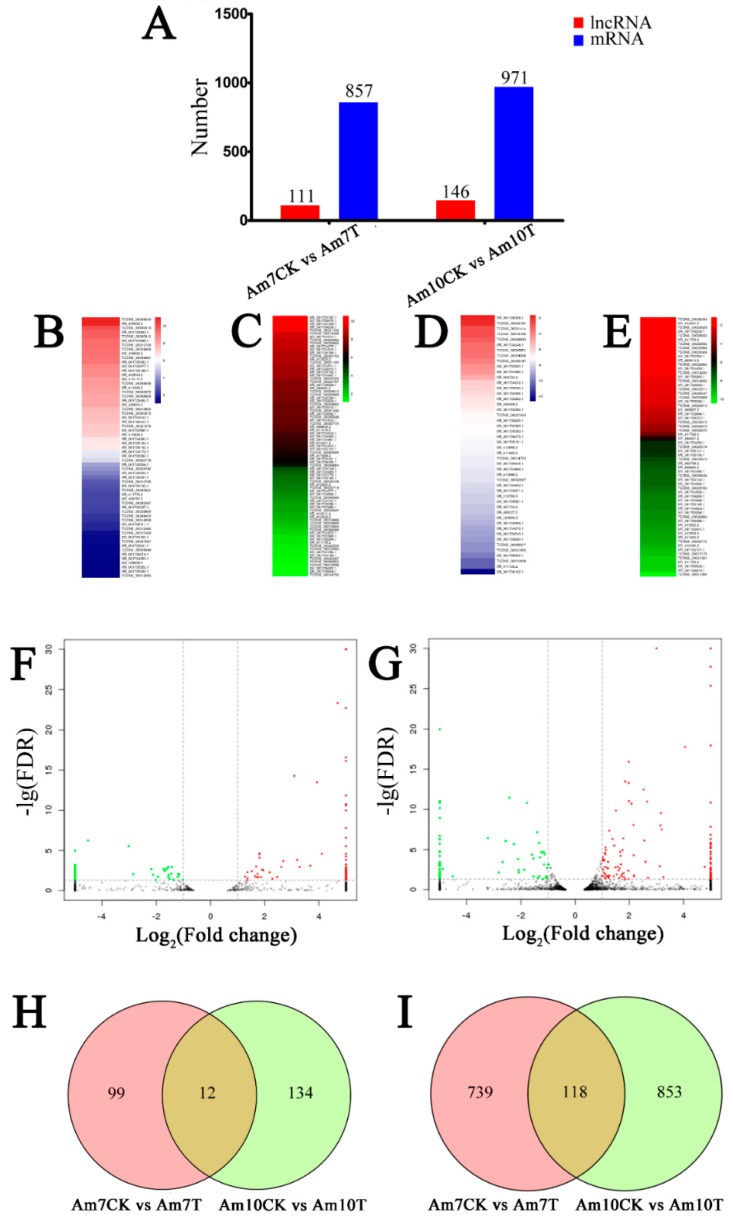
Differential expression patterns of *A. m. ligustica* lncRNAs and mRNAs in *N. ceranae*-infected midguts compared with normal midguts. (**A**) number of differentially expressed lncRNAs (DElncRNAs) and differentially expressed genes (DEGs). (**B**,**C**) expression clustering of up- and down-regulated lncRNAs in Am7CK vs. Am7T. (**D**,**E**) expression clustering of up- and down-regulated lncRNAs in Am10CK vs. Am10T. (**F**,**G**) volcano plots of DEGs in Am7CK vs. Am7T and Am10CK vs. Am10T. (**H**) venn diagram of DElncRNAs in Am7CK vs. Am7T and Am10CK vs. Am10T. (**I**) venn diagram of DEGs in Am7CK vs. Am7T and Am10CK vs. Am10T.

Our main objective was to identify candidate *N. ceranae*-responsive lncRNAs involved in *A. m. ligustica* workers’ midguts. In total, 111 lncRNAs were differentially expressed in Am7CK vs. Am7T, including 62 up-regulated and 49 down-regulated lncRNAs (Figure 3A, Table S3); while in Am10CK vs. Am10T, 146 DElncRNAs including 82 up-regulated and 64 down-regulated lncRNAs were identified (Figure 3A, Table S4). The expression clustering of DElncRNAs in Am7CK vs. Am7T and Am10CK vs. Am10T was further conducted, and the result showed that various DElncRNAs have differential expression levels (Figure 3B–E). Among them, TCONS_00037745 and TCONS_00029069 were the most up-regulated, while XR_001706167.1 and TCONS_00011956 were the most down-regulated. In addition, 857 and 971 mRNAs showed differential expression levels in *N. ceranae*-treated groups compared with control groups (472 up-regulated and 385 down-regulated mRNAs in Am7CK vs. Am7T; 611 up-regulated and 360 down-regulated mRNAs in Am10CK vs. Am10T) (Figure 3A). Similar differential expression trends of mRNAs were observed in the volcano plots (Figure 3F,G). The numbers of DEGs and DElncRNAs among two comparison groups demonstrated an increase as the *N. ceranae* stress progressed (Figure 3A).

Moreover, Venn analyses showed that 12 DElncRNAs were shared by Am7CK vs. Am7T and Am10CK vs. Am10T, while the numbers of unique DElncRNAs in the two comparison groups were 99 and 134, respectively (Figure 3H); 118 DEGs were common between Am7CK vs. Am7T and Am10CK vs. Am10T, while 739 and 853 were specific in the two comparison groups, respectively (Figure 3I).

### 3.5. Functional Investigation of N. ceranae-Responsive lncRNAs in A. m. ligustica Workers’ Midguts

Previous studies proved that lncRNAs could regulate the expression of target genes via chromatin remodeling, control of transcription initiation, and posttranscriptional processing [74,75]. LncRNAs can regulate target gene expression by acting in *cis* on neighboring loci [74]. In the current work, we investigated the *cis* role of lncRNAs by screening the protein-coding genes as potential lncRNA *cis*-regulatory targets in the regions located 10-kb upstream and downstream of all identified lncRNAs for prediction of their functional roles. GO analyses suggested the putative target genes of DElncRNAs in Am7CK vs. Am7T, which were annotated as 10 biological process-associated terms such as metabolic process (17) and cellular process (17), 10 molecular function-related terms such as binding (26) and catalytic activity (23), and 11 cellular component-connected terms such as cell (8) and membrane (6); similarly, the targets of DElncRNAs in Am10CK vs. Am10T were annotated with 14 biological process-associated terms (e.g., single-organism process, localization, and biological regulation), 11 molecular function-related terms (e.g., transporter activity, signal transducer activity, and molecular transducer activity), and 13 cellular component-connected terms (e.g., organelle, macromolecular complex, and extracellular region). The top 15 significant GO terms are presented in Tables S5 and S6. KEGG pathway enrichment analyses concluded that 27 neighboring genes of DElncRNAs in Am7CK vs. Am7T were enriched in 47 pathways associated with organismal systems (seven; e.g., immune system and aging), metabolisms (22; e.g., carbon metabolism and purine metabolism), genetic information processing (10; e.g., DNA replication and ribosome), environmental information processing (four; e.g., signal transduction and membrane transport), and cellular processes (peroxisome and lysosome); comparatively, 41 neighboring genes of DElncRNAs in Am10CK vs. Am10T were enriched in 50 pathways, including 30 related to material and energy metabolisms such as starch and sucrose metabolism (TCONS_00010783, XM_394494.6), and glycerolipid metabolism (XM_006562913.2), and cellular pathways such as ubiquitin-mediated proteolysis (TCONS_00027323, XM_006570714.2), endocytosis (XM_016915026.1, XM_016917619.1), and lysosome (XM_016914010.1). The top 15 significantly enriched pathways are shown in Tables S7 and S8.

In addition, lncRNAs can regulate target gene expression by operating in *trans* [76]. Therefore, we also investigated the *trans* role of lncRNAs by screening the protein-coding genes as potential lncRNA *trans*-regulatory targets of all identified lncRNAs for prediction of their functional roles. GO analyses suggested the putative target genes of DElncRNAs in Am7CK vs. Am7T, which were annotated as 12 biological process-associated terms such as metabolic process (29) and cellular process (22), eight molecular function-related terms such as binding (23) and catalytic activity (23), and six cellular component-connected terms such as membrane (12) and membrane part (10); similarly, the targets of DElncRNAs in Am10CK vs. Am10T were annotated with 17 biological process-associated terms (e.g., cellular process, metabolic process, and single-organism process), nine molecular function-related terms (e.g., binding, catalytic activity, and molecular transducer activity), and 13 cellular component-connected terms (e.g., membrane, cell, and cell part). The top 15 significant GO terms are presented in Tables S9 and S10. KEGG pathway analyses concluded that 20 target genes of DElncRNAs in Am7CK vs. Am7T were enriched in 60 pathways associated with organismal systems (19; e.g., endocrine system and immune system), metabolisms (14; e.g., global and overview maps and energy metabolism), genetic information processing (five; e.g., folding, sorting and degradation, and translation), environmental information processing (14; e.g., signal transduction, and signaling molecules and interaction), and cellular processes (8; e.g., cell growth and death, and cellular community-eukaryotes); comparatively, 55 target genes of DElncRNAs in Am10CK vs. Am10T were enriched in 136 pathways, including metabolic pathways, phototransduction, and gastric acid secretion. The top 15 significantly enriched pathways are shown in Tables S11 and S12.

### 3.6. Discovery of A. m. ligustica lncRNAs as miRNA Precursors and ceRNAs

LncRNA loci that overlapped with miRNA loci on the same strand were regarded as the miRNA precursors [77]. To determine whether lncRNAs are in fact precursors of miRNAs, the lncRNA sequences were compared with the miRNA sequences obtained from miRBase. The result demonstrated that 27 lncRNAs harbored eight complete known miRNA precursors (Table S13); in addition, the secondary structures of lncRNA transcripts suggested that many known and novel lncRNAs contained a stable hairpin structure for miRNA precursors. For example, TCONS_00019779 harbored ame-mir-927a (Figure S5), while TCONS_00036128 harbored ame-mir-1-1 and ame-mir-750. Additionally, another 513 *A. m. ligustica* lncRNAs were predicted to be precursors of 2257 novel miRNAs (Table S14).

**Figure 4:**
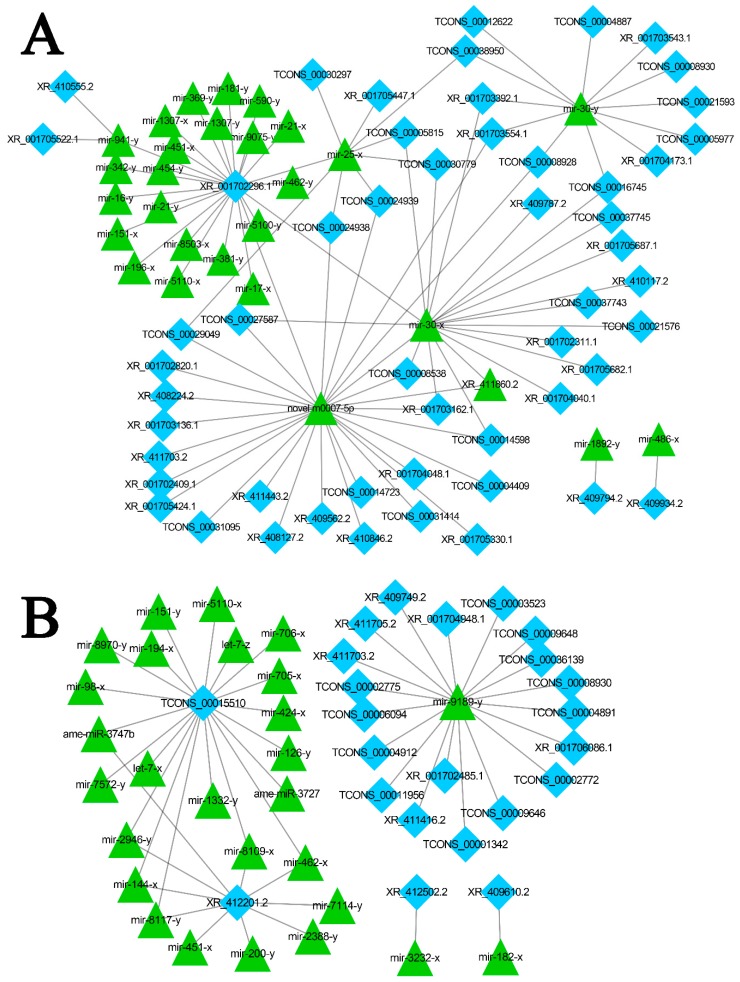
Competitive endogenous RNA (CeRNA) networks of DElncRNAs in *N. ceranae*-infected and normal midguts of *A. m. ligustica* workers. (**A**) ceRNA networks of DElncRNAs in Am7CK vs. Am7T. (**B**) ceRNA networks of DElncRNAs in Am10CK vs. Am10T. Diamonds indicate lncRNAs and triangles indicate miRNA.

The competitive endogenous RNAs (ceRNAs) including mRNAs and lncRNAs containing shared miRNA response elements (MREs), and they can compete for miRNA binding [78]. LncRNAs may bind miRNAs as ceRNAs, thereby functioning as miRNA sponges [79]. The lncRNA-miRNA interaction can be examined using traditional miRNA target prediction methods [80,81]. Here, we analyzed the 6353 lncRNA transcripts that may harbor MREs for ceRNA networks [78] using miRanda [62], PITA [82], and RNAhybrid [63,64]. As shown in Figure 4, complex ceRNA networks of *A. m. ligustica* DElncRNAs and their target miRNAs were visualized using Cytoscape. A total of 106 DElncRNAs in Am7CK vs. Am7T were detected to target 83 *A. m. ligustica* miRNAs (Table S15). Of these, some DElncRNAs were targeted by more than one miRNAs. For example, XR_001702296.1 and TCONS_00030779 could be targeted by 23 and 20 miRNAs, respectively; additionally, some DElncRNAs were targeted by only one miRNA, such as XR_409934.2, XR_409794.2, and XR_001703543.1. Meanwhile, several lncRNAs had the same target miRNA. For example, XR_410555.2, XR_001705522.1, and TCONS_00030779 can target mir-941-y; as many as 28 lncRNAs including XR_001703554.1 and TCONS_00031414 could target novel-m0007-5p. In the Am10CK vs. Am10T comparison group, 143 DElncRNAs were predicted to be targets of 107 miRNAs (Table S16). Similarly, a portion of the DElncRNAs such as XR_412201.2 and TCONS_00015510 were targeted by several miRNAs, while some (e.g., XR_412502.2 and XR_409610.2) had only one target miRNA. In addition, some DElncRNAs including XR_001706086.1, XR_001702485.1, and TCONS_00036139, were targeted by the same miRNA (mir-9189-y).

### 3.7. DElncRNA-miRNA-mRNA Regulatory Networks in A. m. ligustica Workers’ Midguts Invaded by N. ceranae

**Figure 5:**
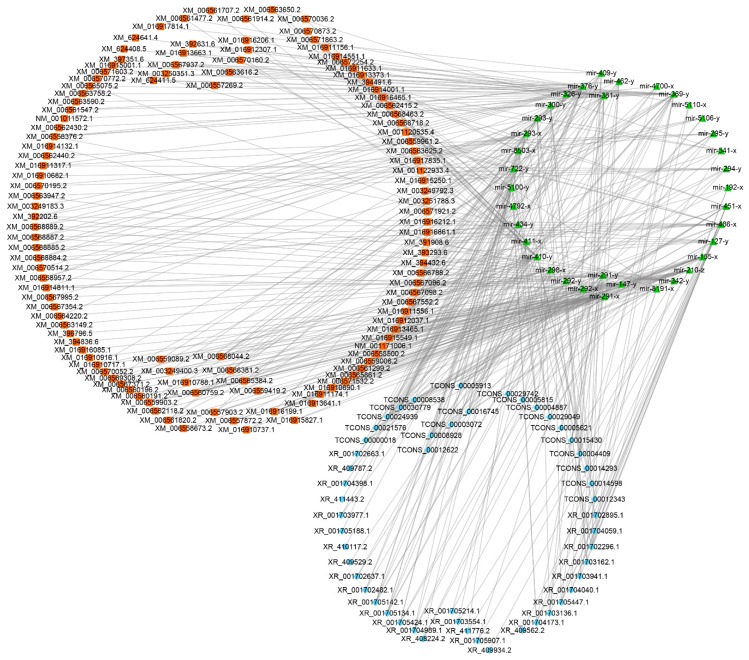
DElncRNA-miRNA-mRNA regulatory networks of up-regulated lncRNAs in Am7CK vs. Am7T. Diamonds indicate lncRNAs, triangles indicate miRNAs, and rectangles indicate mRNAs.

**Figure 6:**
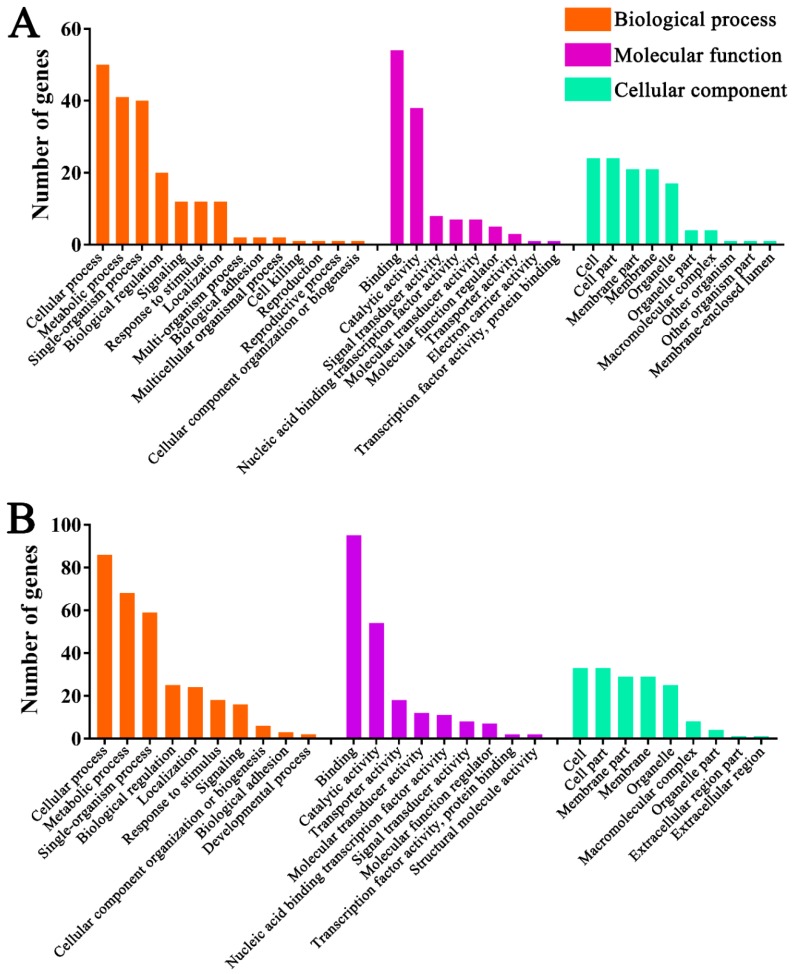
Gene ontology (GO) categorizations of target genes of DElncRNA-targeted miRNAs in Am7CK vs. Am7T (**A**) and Am10CK vs. Am10T (**B**).

To further investigate the roles of DElncRNAs, target mRNAs of DElncRNA-targeted miRNAs were predicted using miRanda [62], RNAhybrid [63,64] and TargetFinder [65]. In total, 278 (Table S17) and 365 target mRNAs (Table S18) were observed in Am7CK vs. Am7T and Am10CK vs. Am10T. DElncRNA-miRNA-mRNA regulatory networks were constructed with Cytoscape, and it was discovered that DElncRNAs, target miRNAs of DElncRNAs, and target mRNAs of DElncRNA-targeted miRNAs formed even more complex networks (Figure 5, Figures S6–S8). GO categorizations demonstrated that target genes in Am7CK vs. Am7T were involved in 14 biological process-related terms including cellular process, metabolic process, and biological regulation; nine molecular function-related terms including binding, catalytic activity, and molecular function regulator; 10 cellular component-related terms including cell, membrane, and organelle (Figure 6A, Table S19); while target genes in Am10CK vs. Am10T were associated with 28 GO terms, which also include the abovementioned terms (Figure 6B, Table S20). Moreover, we found that 12 and 18 target genes in Am7CK vs. Am7T and Am10CK vs. Am10T were engaged in response to stimulus; 12 and 16 target genes were associated with signaling, respectively (Figure 6).

**Figure 7:**
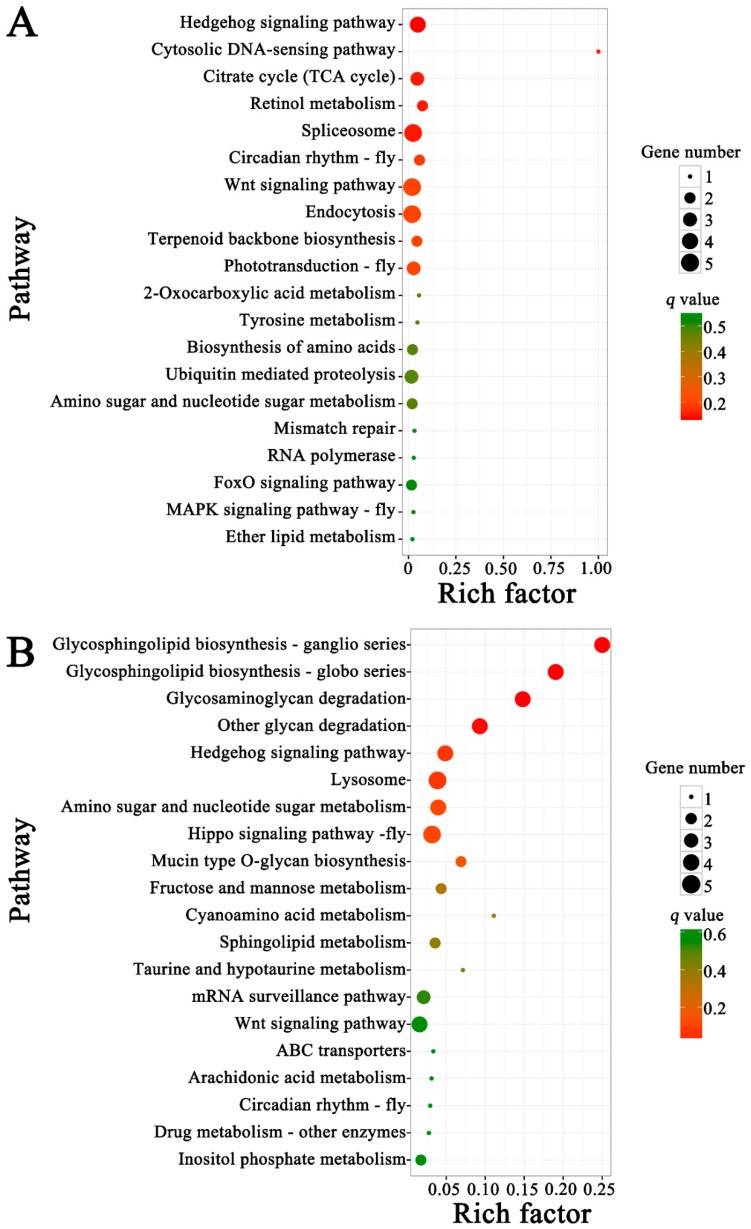
Kyoto Encyclopedia of Genes and Genomes (KEGG) pathway enrichment analyses of target genes of DElncRNA-targeted miRNAs in Am7CK vs. Am7T (**A**) and Am10CK vs. Am10T (**B**).

Further, pathway analyses demonstrated target mRNAs in Am7CK vs. Am7T were enriched in 39 pathways, including 19 metabolism-related pathways such as biosynthesis of amino acids and oxidative phosphorylation, eight genetic information processing-related pathways such as transcription and translation; six signal transduction-related pathways such as the Wnt signaling pathway and Hippo signaling pathway (Figure 7A, Table S21); while target genes in Am10CK vs. Am10T were involved in 45 pathways; among them, 23, seven, and seven were relevant to metabolism, genetic information processing, and signal transduction, respectively (Figure 7B, Table S22). Interestingly, target mRNAs in both Am7CK vs. Am7T and Am10CK vs. Am10T were enriched in cellular immunity-related pathways including endocytosis, phagosome, and ubiquitin-mediated proteolysis; however, only five target genes in Am10CK vs. Am10T were associated with lysosome, no genes in Am7CK vs. Am7T were found to be associated with any humoral immune pathway (Figure 7).

### 3.8. Validation of DElncRNAs byRT-qPCR

**Figure 8:**
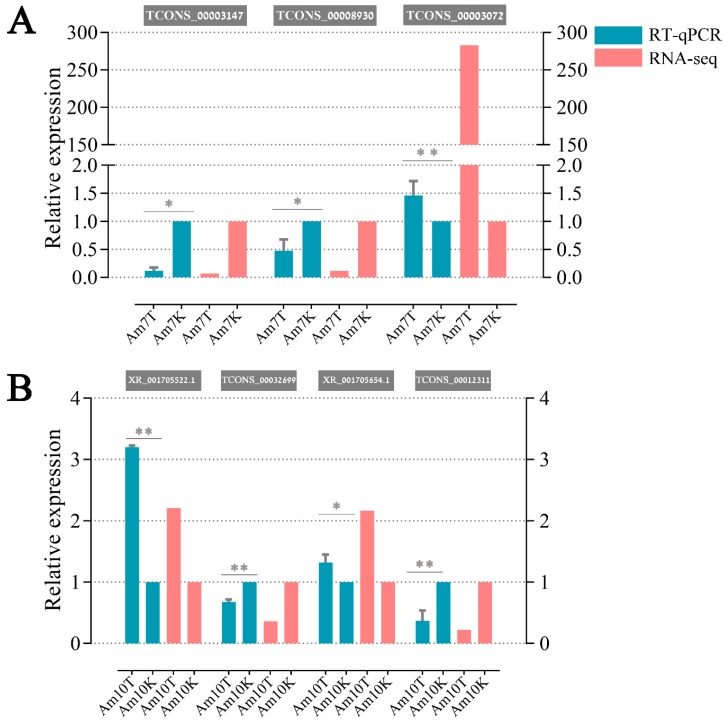
Validation of the differential expression patterns of *A. m. ligustica* lncRNAs via RT-qPCR (**A**,**B**). *t*-Test: * *p* < 0.05; ** *p* < 0.01.

The results indicated that the expression patterns of seven DElncRNAs were in agreement with the RNA-seq results (Figure 8), confirming the transcriptome sequencing data and differential expression patterns of lncRNAs.

## 4. Discussion

In the last decade, lncRNA has become a worldwide research hotspot attracting increasing attention, and the overwhelming majority of lncRNA studies have been performed in mammals and plants, especially in model species such as human [19] and *Arabidopsis* [30]. However, studies on insect lncRNAs are still in the initial stage. Recently, a series of lncRNAs was been discovered in several insect species, including *Drosophila melanogaster* [83], *Anopheles gambiae* [35], *Nilaparvata lugens* [84], and *Bombyx mori* [36]. An array of studies focused on mRNA expression pattern in honeybee after *N. ceranae* infection were previously conducted [67,85,86], which provide valuable information for researchers interested in this domain. Recently, Evans group performed several studies on western honey bee and *N. ceranae* miRNAs [87,88,89], opening an attracting research direction for honeybee-microspodian interaction. However, little is known about the profiling and role of other ncRNAs such as lncRNAs and circular RNAs involved in interactions between honeybee and microspodian. More recently, our group identified lncRNAs and circRNAs in *N. ceranae* based on bioinformatics and preliminarily verified their expressions using molecular method [33,90]. Here, based on these previous works, we for the first time used rRNA removal and strand-specific RNA sequencing to systematically characterize and identify lncRNAs involved in the responses of *A. m. ligustica* to *N. ceranae* infection. In comparison with polyA enrichment sequencing, this method offers the obvious advantage of allowing non-polyA transcripts to be gained [91]; hence, strand information of lncRNAs was also included in our sequencing data, allowing us to distinguish sense transcripts from antisense transcripts. Considering that different kinds of lncRNAs may serve their functions in various manners, a detailed categorization of lncRNAs would facilitate further understanding of their multiple functions [21]. In this work, 4749 conserved lncRNAs and 1604 novel lncRNAs were predicted from normal and *N. ceranae*-infected midguts of *A. m. ligustica* workers, which offered a relatively robust list of potential lncRNAs for *A. m. ligustica*. This set of lncRNAs will be beneficial for functional genomics research and complementing the reference genome annotation of *Apis mellifera*. In our study, we detected that most lncRNAs were expressed at relatively low levels following an FPKM cutoff, and thus, lncRNAs with low expression may be ignored. Higher RNA-seq coverage can partially overcome this problem [92]. Considering that lncRNAs are often expressed in a tissue- or development-specific manner [93], it is believed that the identified lncRNAs occupied just a fraction of the total lncRNAs in *A. m. ligustica*, and that more lncRNAs may be discovered using different castes, various organs and tissues, and organs and tissues under different stresses.

In our study, these *A. m. ligustica* lncRNAs were found to share some features with their counterparts in other species, including relatively short lengths, low exon numbers, and relatively low expression [94,95,96], indicating that these characteristics are common for lncRNAs in most species [35,36,83,84]. In vertebrates, lncRNAs have poor sequence conservation compared to protein-coding genes, making it quite difficult to predict the functions of lncRNAs simply based on their nucleotide sequences [18,19,97,98]. For example, less than 6% of zebrafish lncRNAs exhibited sequence conservation with human or mouse lncRNAs. Previous studies have demonstrated that the sequence conservation of lncRNAs between human and other species was only approximately 12% [99,100]. In the present study, the identified lncRNAs were compared against the NCBI Nr and NONCODE databases, but no highly similar sequences were found (data not shown), indicative of a lack of sequence conservation of *A. m. ligustica* lncRNAs.

Under abiotic and biotic stresses, lncRNAs could be used to regulate gene expression in multiple ways. For example, lncRNAs play pivotal roles in controlling the stress-response in plants including *Populus* [101] and wheat [31]. Jayakodi et al. identified 15 lincRNAs showing significant differential expression between sacbrood virus (SBV)-infected and uninfected *Apis cerana*, and further confirmed the expression of 11 lincRNAs using RT-qPCR [38]. In this work, the expression levels of 111 and 146 lncRNAs were observed to be significantly altered in *N. ceranae*-infected midguts of *A. m. ligustica* workers at 7 dpi and 10 dpi, which suggested that the expression levels of some lncRNAs were affected by *N. ceranae* infection.

The functions of lncRNAs are highly diverse; however, few known lncRNAs had functional annotations. To predict the functional roles of lncRNAs, their correlated protein-coding genes and associated biological pathways are surveyed to gain useful information [28]. The *cis* effect is defined as the regulatory action of lncRNAs on genes located upstream or downstream, which has been proven to be a common mechanism [102]. Here, to further investigate the roles of lncRNAs involved in the *A. m. ligustica* response to *N. ceranae*, the potential function of the DElncRNAs was predicted using the *cis* method. GO classifications suggested that the targets of DElncRNAs in Am7CK vs. Am7T and Am10CK vs. Am10T were respectively engaged in 31 and 38 functional terms, and among them, binding-associated activity was the most abundant in both comparison groups. LncRNAs are key regulators of various biological functions, but the related mechanisms are not fully understood. One of the regulatory mechanisms is based on interactions between different biological macromolecules such as RNA-RNA and RNA-DNA interactions [103]. Therefore, it is believed that *A. m. ligustica* lncRNAs were likely to participate in the *N. ceranae*-response via these interactions. However, it should be noted that the lines of evidence presented here are indirect, and further experiments are needed to verify the interaction among these lncRNAs and their targets. Furthermore, pathway analyses demonstrated that the DElncRNAs were involved in regulating 47 and 50 pathways. *N. ceranae* is a mitochondria-driven species that has a high dependency on host ATP, leading the honeybee to increase sucrose needs and glycometabolism-related gene expression [67,104]. Similarly, in this study, neighboring genes of DElncRNAs were enriched in multiple pathways associated with glucose metabolism, including galactose, starch and sucrose, fructose and mannose, and other polysaccharide metabolisms. Intriguingly, we detected that the number of glycometabolism-associated pathways in host midgut at 10 dpi (4) was more than that in midgut at 7 dpi (2), suggestive of the participation of DElncRNAs in glycometabolism in host responses to *N. ceranae*. This result implied that, with the prolonged stress duration, *A. m. ligustica* worker needed to tranform various sugars in food into ATP as much as possible to offset the energy stolen by *N. ceranae*. Previous studies showed that *N. ceranae* could inhibit honeybee cell apoptosis to provide itself with enough time for proliferation [50,105,106,107]. Compared with RNA viruses and *Varroa destructor*, only *N. ceranae* or *Nosema apis* can regulate apoptosis-related genes of bees [108]. However, it is still unknown whether honeybee lncRNAs participate in regulation of cell apoptosis and honeybee-*N. ceranae* interaction. In our study, we observed two apoptosis-inducing related genes XM_006560835.2 (up-regulation at 7 dpi, while down-regulation at 10 dpi) and XM_625032.5 (down-regulation at 7 dpi and 10 dpi) located up- and down-stream of DElncRNAs, which indicated that *N. ceranae* inside the host cells might adopt a lncRNA-mediated strategy to affect host cell apoptosis. However, the underlying mechanism remain unknown here and requires further effort. When cuticles and peritrophic membranes are breached, the pathogenic microorganism encounters a set of efficient cellular and humoral defenses including encapsulation, melanization, phagocytosis, enzymatic degradation of pathogens as well as secretion of antimicrobial peptides [109,110]. In honeybees, phagocytosis and encapsulation are the two most common defense mechanisms against fungal invasion [109,110]. In this current work, four, two, two, and two source genes of DElncRNAs were observed to be enriched in ubiquitin-mediated proteolysis, endocytosis, lysosome, and metabolism of xenobiotics by cytochrome P450. This result suggested that these cellular immune pathways may be regulated by DElncRNAs during the host *N. ceranae*-response process. Taken together, these results demonstrated that the corresponding DElncRNAs were likely to be specific regulators during *A. m. ligustica* responses to *N. ceranae* stress, and that these DElncRNAs may participate in the *N. ceranae*-response via interactions with their source genes. Some evidences suggest that lncRNAs are capable of exerting regulatory roles via action in *trans* [76]. In this current work, the co-expressed genes of DElncRNAs in Am7CK vs. Am7T and Am10CK vs. Am10T were predicted and subjected to further analyses. The results demonstrated these target genes were involved in a series of functional terms associated with biological process, molecular function, and cellular component, as well as a number of pathways related to material and energy metabolisms as well as cellular activities. Collectively, these results suggested that DElncRNAs can participate in host response to *N. ceranae* infection through *cis* and *trans* approaches, supporting the various action modes of lncRNAs.

In contrast with small ncRNAs, our understanding of the functions and regulatory mechanisms of lncRNAs is rather limited. Another manner for lncRNAs to exert their regulatory functions is to produce or interact with small RNAs [111,112]. In this work, 27 lncRNAs were detected to contain eight known miRNA precursors; additionally, 513 lncRNAs harboring 2257 novel miRNA precursors were discovered. The results indicated that some *A. m. ligustica* lncRNAs could be processed into miRNAs to exert their functions. We inferred that lncRNAs might be an important resource for identifying novel miRNAs. Some lncRNAs containing MREs have been proven to communicate with and regulate corresponding miRNA target genes via specifically competing for shared miRNAs [113,114]. Investigation of well-established miRNAs may help researchers to understand the functions of associated lncRNAs. In the present study, we detected that 106 and 143 DElncRNAs respectively interact with 83 and 107 miRNAs in Am7CK vs. Am7T and Am10CK vs. Am10T. Within the complex lncRNA-miRNA interaction networks, some DElncRNAs can link to the same miRNA, while some DElncRNAs could be targeted by several miRNAs (Figure 4). Several miRNAs were deeply studied such as miR-25 [115] and miR-30 [116]. By using miR-25 mimic and inhibitor, Hua et al. found miR-25 can reduce the expression of MALAT1 (metastasis-associated with lung adenocarcinoma transcript 1) as a tumor suppressor in nasopharyngeal carcinoma [115]. As shown in the study conducted by Xie et al., upregulating the expression level of miR-30a could reduce CD73′s (ecto-5′-nucleotidase) expression, thereby restraining the proliferation ability of CRC (colorectal cancer) cells and promoting cellular apoptosis as a tumor suppressor [116]. In this work, it was detected that miR-25-x (homologous to miR-25) was the target of 11 DElncRNAs, while miR-30-x and miR-30-y (homologous to miR-30a) were targeted by 19 and 15 DElncRNAs. We inferred that these *A. m. ligustica* DElncRNAs might suppress *N. ceranae* via crosstalk with miR-25-x, miR-30-x, and miR-30-y. However, the binding of DElncRNA and miRNAs is limited to bioinformatic prediction and more experimental evidence is required.

DElncRNA-miRNA-mRNA regulation networks were further constructed and analyzed to explore the potential roles of the dysregulated lncRNAs. In this study, 59 up-regulated and 47 down-regulated lncRNAs in Am7CK vs. Am7T, and 55 up-regulated and 88 down-regulated lncRNAs in Am10CK vs. Am10T were involved in regulation networks. Based on GO classifications, we detected that 54 and 95 target genes of DElncRNAs in midguts at 7 dpi and 10 dpi were engaged in binding-associated activities, similar to the finding mentioned above. In addition, we observed that 12 target genes and one target gene of DElncRNAs in Am7CK vs. Am7T were involved in response to stimulus and cell killing, while 18 targets of DElncRNAs in Am10CK vs. Am10T were enriched in response to stimulus, indicative of the involvement of corresponding DElncRNAs in host defense against *N. ceranae*. Moreover, pathway analyses demonstrated that a variety of target genes of DElncRNAs in both comparison groups were enriched in material metabolism-associated pathways, such as carbohydrate metabolism (e.g., citrate cycle and galactose metabolism), lipid metabolism (e.g., glycerolipid metabolism and glycerophospholipid metabolism), and nucleotide metabolism (e.g., pyrimidine metabolism and purine metabolism). Intriguingly, only one target (XM_006572254.2) of three DElncRNAs (XR_001702895.1, XR_411776.2, and XR_001704989.1) in midguts at 7 dpi was detected to be associated with oxidative phosphorylation, an important energy metabolism pathway; however, there was no enriched target gene of DElncRNAs in midguts 10 dpi. This indicates the participation of the aforementioned DElncRNAs in regulating host energy metabolism during the early stage of *N. ceranae* stress. Huang et al. investigated western honey bee miRNAs daily across a full 6-day reproduction cycle of *N. ceranae* and identified 17 host DEmiRNAs [81]. However, when compared these previously reported 17 miRNAs and the target miRNAs of DElncRNAs observed in this work, no shared miRNAs were found, which implied that miRNA-mediated and lncRNA-mediated regulatory networks may be two different strategies employed by western honeybee to response to *N. ceranae* infection. Different sample age and inoculation dose used in this work and previous study may be factors responsible for the different findings. Interestingly, we detected that the target genes of DEmiRNAs predicted in previous study were involved in oxidative phosphorylation, which was also enriched by the targets of DElncRNA-targeted miRNAs discovered in our study. This indicates that both miRNAs and lncRNAs affected the energy metabolism pathway during host response to *N. ceranae* invasion. Additionally, target genes of DElncRNA-targeted miRNAs in Am7CK vs. Am7T were engaged in three cellular immune pathways such as endocytosis (5), phagosome (1), and ubiquitin-mediated proteolysis (3); one humoral immune pathway (MAPK signaling pathway, 1) was also engaged. While, only three (endocytosis, ubiquitin-mediated proteolysis, and MAPK) of these four immune pathways were enriched in Am10CK vs. Am10T. Moreover, five target genes of miRNAs targeted by DElncRNA in midgut at 10 dpi were enriched in lysosome pathway, similar to previous finding [86]. The result suggests that host miRNAs and lncRNAs can regulate this cellular immune pathway during *N. ceranae*-response. Collectively, these results demonstrated that corresponding DElncRNAs may play specific roles in the regulation of the abovementioned cellular and humoral immune pathways. We speculated that DElncRNAs can regulate the expression of target genes mediated by interactional miRNAs during the *N. ceranae*-response of *A. m. ligustica*.

## 5. Conclusions

In summary, we identified 4749 known lncRNAs and 1604 novel lncRNAs in the midguts of *A. m. ligustica* workers, and showed that 111 and 146 lncRNAs were *N. ceranae*-responsive in midguts at 7 dpi and 10 dpi, respectively. These results suggest that the expression of host lncRNAs was significantly altered by *N. ceranae* infection; a portion of the DElncRNAs were likely to participate in *N. ceranae*-response processes by regulating gene expression in *cis* and *trans* fashion or by serving as miRNA precursors or ceRNAs, and thus may be potential new therapeutic targets for microsporidiosis. Our data provide a rich genetic resource for further investigation of the functional roles of lncRNAs involved in the *A. m. ligustica* responses to *N. ceranae* infection, but also a foundation for revealing the underlying molecular mechanisms. Furthermore, this work offers novel insights into understanding host-pathogen interactions during honeybee microsporidiosis.

## Figures and Tables

**Table 1:** Quality control of transcriptome data.

Sample	Raw Reads	Clean Reads (%)	Q20 (%)	Q30 (%)
Am7CK1	160,844,082	160,049,106 (99.51)	23,340,144,349 (97.41)	22,521,956,996 (94.00)
Am7CK2	129,878,194	129,283,918 (99.54)	18,891,245,674 (97.56)	18,239,412,915 (94.19)
Am7CK3	113,683,898	113,165,446 (99.54)	16,535,666,991 (97.52)	15,943,589,998 (94.03)
Am7T1	152,323,278	151,668,484 (99.57)	22,161,043,664 (97.55)	21,387,125,499 (94.15)
Am7T2	200,417,896	199,313,090 (99.45)	28,948,504,448 (97.11)	27,829,913,730 (93.35)
Am7T3	126,667,596	126,053,962 (99.52)	18,386,919,122 (97.38)	17,719,616,862 (93.85)
Am10CK1	160,537,248	159,765,346 (99.52)	23,262,715,888 (97.27)	22,443,038,732 (93.84)
Am10CK2	149,230,808	148,494,716 (99.51)	21,633,348,548 (97.28)	20,852,891,752 (93.77)
Am10CK3	131,386,354	130,619,802 (99.42)	18,959,297,638 (96.98)	18,248,516,385 (93.34)
Am10T1	249,473,666	248,333,982 (99.54)	36,162,922,479 (97.32)	34,857,597,435 (93.81)
Am10T2	208,589,832	207,574,770 (99.51)	30,251,988,213 (97.34)	29,139,831,253 (93.77)
Am10T3	173,097,006	172,166,682 (99.46)	25,113,348,781 (97.38)	24,175,449,594 (93.74)

## Supplementary Materials

The following are available online at https://www.mdpi.com/2075-4450/10/8/245/s1, Figure S1. Experimental inoculation of an *A. m. ligustica* worker with *N. ceranae* spores. (A) Microscopic observation of purified spores of *N. ceranae* (400×). (B) Artificial inoculation of an *A. m. ligustica* worker with *N. ceranae* spores, Figure S2. Detection of RNA viruses and *N. ceranae* in newly emerged *A. m. ligustica* workers, Figure S3. Bioinformatic pipeline for prediction of *A. m. ligustica* lncRNAs, Figure S4. Pearson correlation coefficients between different biological replicas within every control and treatment groups, Figure S5. Secondary structure of TCONS_00019779 that harbors ame-mir-927a, Figure S6. DElncRNA-miRNA-mRNA regulatory networks of down-regulated lncRNAs in Am7CK vs. Am7T, Figure S7. DElncRNA-miRNA-mRNA regulatory networks of up-regulated lncRNAs in Am10CK vs. Am10T, Figure S8. DElncRNA-miRNA-mRNA regulatory networks of down-regulated lncRNAs in Am10CK vs. Am10T, Table S1 Primers for RT-PCR confirmation of honeybee viruses and *N. ceranae*, Table S2 Primers for RT-qPCR validation performed in this study, Table S3 Detailed information of DElncRNAs in the Am7CK vs. Am7T, Table S4 Detailed information of DElncRNAs in the Am10CK vs. Am10T, Table S5 Top 15 GO categories enriched by *cis*-regulatory target genes of DElncRNAs in Am7CK vs. Am7T, Table S6 Top 15 GO categories enriched by *cis*-regulatory target genes of DElncRNAs in Am10CK vs. Am10T, Table S7 Top 15 pathways enriched by *cis*-regulatory target genes of DElncRNAs in Am7CK vs. Am7T, Table S8 Top 15 pathways enriched by *cis*-regulatory target genes of DElncRNAs in Am10CK vs. Am10T, Table S9 Top 15 GO categories enriched by *trans*-regulatory target genes of DElncRNAs in Am7CK vs. Am7T, Table S10 Top 15 GO categories enriched by *trans*-regulatory target genes of DElncRNAs in Am10CK vs. Am10T, Table S11 Top 15 pathways enriched by *trans*-regulatory target genes of DElncRNAs in Am7CK vs. Am7T, Table S12 Top 15 pathways enriched by *trans*-regulatory target genes of DElncRNAs in Am10CK vs. Am10T, Table S13 Detailed information of 27 *A. m. ligustica* lncRNAs harboring 8 complete known miRNA precursors, Table S14 Detailed information of 513 *A. m. ligustica* lncRNAs harboring 2257 novel miRNA precursors, Table S15 Pairs of DElncRNAs and corresponding target miRNAs in Am7CK vs. Am7T, Table S16 Pairs of DElncRNAs and corresponding target miRNAs in Am10CK vs. Am10T, Table S17 Pairs of DElncRNA-targeted miRNAs and corresponding target mRNAs in Am7CK vs. Am7T, Table S18 Pairs of DElncRNA-targeted miRNAs and corresponding target mRNAs in Am10CK vs. Am10T, Table S19 Summary of GO classifications of target genes of DElncRNA-targeted miRNAs in Am7CK vs. Am7T, Table S20 Summary of GO classifications of target genes of DElncRNA-targeted miRNAs in Am10CK vs. Am10T, Table S21 Summary of KEGG database annotations of target genes of DElncRNA-targeted miRNAs in Am7CK vs. Am7T, Table S22 Summary of KEGG database annotations of target genes of DElncRNA-targeted miRNAs in Am10CK vs. Am10T.
