# Supplementary material for: Genome-Wide Identification of Long Non-Coding RNAs and Their Regulatory Networks Involved in Apis mellifera ligustica Response to Nosema ceranae Infection
Source: Insects. 2019 Aug 9;10(8):245. doi: 10.3390/insects10080245 (PMC6723323; doi:10.3390/insects10080245)
Supplement: Supplementary file 1 [file insects-10-00245-s001.zip › Supplementary Materials/Figure S8.pdf]

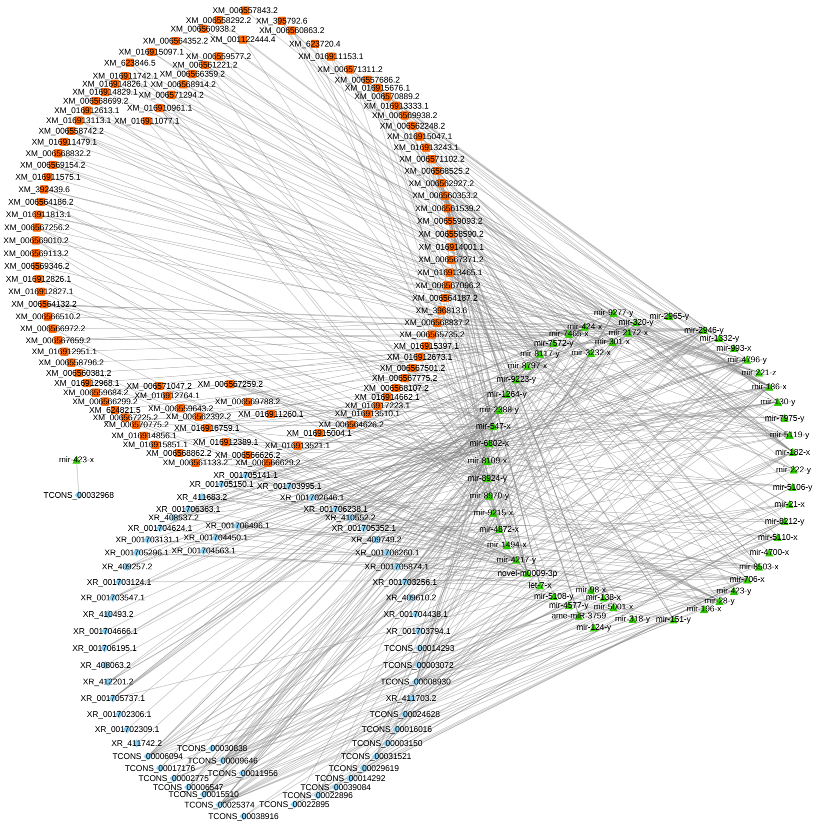

XM_006557843.2
XM_006558292.2
XM_395792.6
XM_006560938.2
XM_006560863.2
XM_006564352.2
XM_001122444.4
XM_623720.4
XM_016915097.1
XM_006559577.2
XM_016911153.1
XM_623846.5
XM_006561221.2
XM_006571311.2
XM_016911742.1
XM_006566359.2
XM_006557686.2
XM_016914826.1
XM_006568914.2
XM_016915676.1
XM_016914829.1
XM_006571294.2
XM_006570889.2
XM_006568699.2
XM_016913333.1
XM_016912613.1
XM_016910961.1
XM_006569938.2
XM_016913113.1
XM_016911077.1
XM_006562248.2
XM_006558742.2
XM_016915047.1
XM_016911479.1
XM_016913243.1
XM_006568832.2
XM_006571102.2
XM_006569154.2
XM_006568525.2
XM_016911575.1
XM_006562927.2
XM_392439.6
XM_006560353.2
XM_006564186.2
XM_006561539.2
XM_016911813.1
XM_006559093.2
XM_006567256.2
XM_006558590.2
XM_006569010.2
XM_016914001.1
XM_006569113.2
XM_006567371.2
XM_006569346.2
XM_016913465.1
XM_016912826.1
XM_006567096.2
XM_016912827.1
XM_006564187.2
XM_006564132.2
XM_396813.6
XM_006566510.2
XM_006568837.2
XM_006566972.2
XM_006565735.2
XM_006567659.2
XM_016915397.1
XM_016912951.1
XM_016912673.1
XM_006558796.2
XM_006567501.2
XM_006560381.2
XM_006567775.2
XM_016912968.1
XM_006571047.2
XM_006567259.2
XM_006568107.2
XM_006559684.2
XM_016912764.1
XM_016914662.1
XM_006566299.2
XM_006569788.2
XM_016917223.1
XM_624821.5
XM_006559643.2
XM_016913510.1
XM_006567225.2
XM_006562392.2
XM_016911260.1
XM_006570775.2
XM_006564626.2
XM_016916759.1
XM_016914856.1
XM_016915004.1
XM_016915851.1
XM_016912389.1
XM_016913521.1
XM_006568862.2
XM_006566626.2
XM_006561133.2
XM_006566629.2
mir-423-x
TCONS_00032968
XR_001705141.1
XR_001705150.1
XR_001703995.1
XR_411683.2
XR_001702646.1
XR_001706363.1
XR_001706238.1
XR_408537.2
XR_410552.2
XR_001704624.1
XR_001706496.1
XR_001705352.1
XR_001703131.1
XR_001704450.1
XR_409749.2
XR_001705296.1
XR_001704563.1
XR_001706260.1
XR_409257.2
XR_001705874.1
XR_001703124.1
XR_001703256.1
XR_001703547.1
XR_409610.2
XR_410493.2
XR_001704438.1
XR_001704666.1
XR_001703794.1
XR_001706195.1
TCONS_00014293
XR_408063.2
TCONS_00003072
XR_412201.2
TCONS_00008930
XR_001705737.1
XR_411703.2
XR_001702306.1
TCONS_00024628
XR_001702309.1
TCONS_00016016
XR_411742.2
TCONS_00003150
TCONS_00030838
TCONS_00006094
TCONS_00031521
TCONS_00009646
TCONS_00017176
TCONS_00029619
TCONS_00011956
TCONS_00002775
TCONS_00014292
TCONS_00006547
TCONS_00039084
TCONS_00015510
TCONS_00022896
TCONS_00025374
TCONS_00022895
TCONS_00038916
mir-9277-y
mir-2965-y
mir-320-y
mir-424-x
mir-2946-y
mir-7465-x
mir-2172-x
mir-1332-y
mir-7572-y
mir-301-x
mir-993-x
mir-8117-y
mir-3232-x
mir-4796-y
mir-8797-x
mir-221-z
mir-9223-y
mir-166-x
mir-1264-y
mir-130-y
mir-2388-y
mir-7575-y
mir-547-x
mir-5119-y
mir-6802-x
mir-182-x
mir-6109-x
mir-222-y
mir-8924-y
mir-5106-y
mir-8970-y
mir-21-x
mir-9215-x
mir-8212-y
mir-4872-x
mir-5110-x
mir-1494-x
mir-4700-x
mir-4217-y
mir-9503-x
novel-m0009-3p
mir-706-x
let-7-x
mir-423-y
mir-98-x
mir-5108-y
mir-138-x
mir-28-y
mir-4577-y
mir-5001-x
mir-106-x
ame-miR-3759
mir-318-y
mir-151-y
mir-124-y
