# Supplementary material for: Genome-Wide Identification of Long Non-Coding RNAs and Their Regulatory Networks Involved in Apis mellifera ligustica Response to Nosema ceranae Infection
Source: Insects. 2019 Aug 9;10(8):245. doi: 10.3390/insects10080245 (PMC6723323; doi:10.3390/insects10080245)
Supplement: Supplementary file 1 [file insects-10-00245-s001.zip › Supplementary Materials/Table S1.docx]

**Table S1** Primers for RT-PCR confirmation of honeybee viruses and *N. ceranae.*

| **Primer name** | **Sequence** | **Product size (bp)** | **Reference** |
| --- | --- | --- | --- |
| DWV-F | TCCATCAGGTTCTCCAATAACGG | 451 | [42] |
| DWV-R | CCACCCAAATGCTAACTCTAACGC |  |  |
| KBV-F | GATGAACGTCGACCTATTGA | 417 | [43] |
| KBV-R | TGTGGGTTGGCTATGAGTCA |  |  |
| IAPV-F | GGTCCAAACCTCGAAATCAA | 840 | [44] |
| IAPV-R | TTGGTCCGGATGTTAATGGT |  |  |
| CBPV-F | AGTTGTCATGGTTAACAGGATACGAG | 455 | [45] |
| CBPV-R | TCTAATCTTAGCACGAAAGCCGAG |  |  |
| BQCV-F | TGGTCAGCTCCCACTACCTTAAAC | 700 | [46] |
| BQCV-R | GCAACAAGAAGAAACGTAAACCAC |  |  |
| ABPV-F | TTATGTGTCCAGAGACTGTATCCA | 900 | [46] |
| ABPV-R | GCTCCTATTGCTCGGTTTTTCGGT |  |  |
| SBV-F | GCACGTTTAATTGGGGATCA | 693 | [44] |
| SBV-R | CAGGTTGTCCCTTACCTCCA |  |  |
| Nc1-F | CAGGATATAGCGATGATTGTG | 112 | This study |
| Nc1-R | AGACCTTCCTCGTAGTGTAT |  |  |
| Nc2-F | AGCACAAGGAGTCGAGCAAA | 100 | [47] |
| Nc2-R | TGCTGCCTCAAATCCTACCT |  |  |
