# Supplementary material for: Genome-Wide Identification of Long Non-Coding RNAs and Their Regulatory Networks Involved in Apis mellifera ligustica Response to Nosema ceranae Infection
Source: Insects. 2019 Aug 9;10(8):245. doi: 10.3390/insects10080245 (PMC6723323; doi:10.3390/insects10080245)
Supplement: Supplementary file 1 [file insects-10-00245-s001.zip › Supplementary Materials/Table S10.docx]

**Table S10** Top 15 GO categories enriched by *trans*-regulatory target genes of DElncRNAs in Am10CK vs Am10T.

| **GO term** | **Number of enriched genes** |
| --- | --- |
| cellular process | 91 |
| binding | 87 |
| metabolic process | 81 |
| catalytic activity | 71 |
| single-organism process | 65 |
| membrane | 37 |
| cell | 36 |
| cell part | 36 |
| membrane part | 35 |
| response to stimulus | 24 |
| biological regulation | 24 |
| localization | 23 |
| regulation of biological process | 23 |
| organelle | 23 |
| signaling | 22 |
