# Supplementary material for: Genome-Wide Identification of Long Non-Coding RNAs and Their Regulatory Networks Involved in Apis mellifera ligustica Response to Nosema ceranae Infection
Source: Insects. 2019 Aug 9;10(8):245. doi: 10.3390/insects10080245 (PMC6723323; doi:10.3390/insects10080245)
Supplement: Supplementary file 1 [file insects-10-00245-s001.zip › Supplementary Materials/Table S11.docx]

**Table S11** Top 15 pathways enriched by *trans*-regulatory target genes of DElncRNAs in Am7CK vs Am7T.

| **Pathway** | **Number of enriched genes** |
| --- | --- |
| Metabolic pathways | 4 |
| Biosynthesis of secondary metabolites | 3 |
| Spliceosome | 3 |
| Dopaminergic synapse | 2 |
| Prolactin signaling pathway | 2 |
| Insulin signaling pathway | 2 |
| Microbial metabolism in diverse environments | 2 |
| Ribosome | 2 |
| PI3K-Akt signaling pathway | 2 |
| Focal adhesion | 2 |
| Neurotrophin signaling pathway | 1 |
| IL-17 signaling pathway | 1 |
| B cell receptor signaling pathway | 1 |
| T cell receptor signaling pathway | 1 |
| Chemokine signaling pathway | 1 |
