# Supplementary material for: Genome-Wide Identification of Long Non-Coding RNAs and Their Regulatory Networks Involved in Apis mellifera ligustica Response to Nosema ceranae Infection
Source: Insects. 2019 Aug 9;10(8):245. doi: 10.3390/insects10080245 (PMC6723323; doi:10.3390/insects10080245)
Supplement: Supplementary file 1 [file insects-10-00245-s001.zip › Supplementary Materials/Table S12.docx]

**Table S12** Top 15 pathways enriched by *trans*-regulatory target genes of DElncRNAs in Am10CK vs Am10T.

| **Pathway** | **Number of enriched genes** |
| --- | --- |
| Metabolic pathways | 21 |
| Phototransduction - fly | 7 |
| Gastric acid secretion | 5 |
| Oxytocin signaling pathway | 5 |
| Biosynthesis of secondary metabolites | 5 |
| Circadian entrainment | 4 |
| Aldosterone synthesis and secretion | 4 |
| Long-term potentiation | 4 |
| Insulin secretion | 4 |
| Adrenergic signaling in cardiomyocytes | 4 |
| Cholinergic synapse | 4 |
| Inflammatory mediator regulation of TRP channels | 4 |
| GnRH signaling pathway | 4 |
| Melanogenesis | 4 |
| Glucagon signaling pathway | 4 |
