# Supplementary material for: Genome-Wide Identification of Long Non-Coding RNAs and Their Regulatory Networks Involved in Apis mellifera ligustica Response to Nosema ceranae Infection
Source: Insects. 2019 Aug 9;10(8):245. doi: 10.3390/insects10080245 (PMC6723323; doi:10.3390/insects10080245)
Supplement: Supplementary file 1 [file insects-10-00245-s001.zip › Supplementary Materials/Table S2.docx]

**Table S2** Primers for RT-qPCR validation performed in this study.

| **Primer F** | **Sequence** | **Primer R** | **Sequence** |
| --- | --- | --- | --- |
| TCONS_00003147-F | AACGAATGGACGACAACAG | TCONS_00003147-R | GATGAGGATTTGAAACCGC |
| TCONS_00008930-F | CCAAGAGCAAGAAGAATGC | TCONS_00008930-R | AGACGGTGAAAGAATCGG |
| TCONS_00003072-F | TCTCTACCCTCTTGGCTACTG | TCONS_00003072-R | TCTCGCTTCTCTTGTTGATG |
| XR_001705522.1-F | CGTTGGTGGTTCAGTTAGCC | XR_001705522.1-R | CGAAATAGAGGTGGAATAAGGG |
| TCONS_00032699-F | AACGCAACAACGGAAGAG | TCONS_00032699-R | TTATCGGACCACGGTTCAC |
| XR_001705654.1-F | ACGAGGCGAGGAATAGAAC | XR_001705654.1-R | GACCAATAATGGAACGAGTGAC |
| TCONS_00012311-F | ATCGCAGTGGAGAGTCAGA | TCONS_00012311-R | ATCCTAAATAAGGGACGCAGAC |
| *Actin*-F | CACTCCTGCTATGTATGTCGC | *Actin*-R | GGCAAAGCGTATCCTTCA |
