# Supplementary material for: Genome-Wide Identification of Long Non-Coding RNAs and Their Regulatory Networks Involved in Apis mellifera ligustica Response to Nosema ceranae Infection
Source: Insects. 2019 Aug 9;10(8):245. doi: 10.3390/insects10080245 (PMC6723323; doi:10.3390/insects10080245)
Supplement: Supplementary file 1 [file insects-10-00245-s001.zip › Supplementary Materials/Table S6.docx]

**Table S6** Top 15 GO categories enriched by *cis*-regulatory target genes of DElncRNAs in Am10CK vs Am10T.

| **GO term** | **Number of enriched genes** |
| --- | --- |
| single-organism process | 27 |
| binding | 27 |
| cellular process | 25 |
| catalytic activity | 23 |
| metabolic process | 20 |
| cell | 15 |
| cell part | 15 |
| localization | 14 |
| membrane | 14 |
| membrane part | 13 |
| biological regulation | 12 |
| organelle | 11 |
| transporter activity | 5 |
| macromolecular complex | 5 |
| signaling | 4 |
