# Supplementary material for: Genome-Wide Identification of Long Non-Coding RNAs and Their Regulatory Networks Involved in Apis mellifera ligustica Response to Nosema ceranae Infection
Source: Insects. 2019 Aug 9;10(8):245. doi: 10.3390/insects10080245 (PMC6723323; doi:10.3390/insects10080245)
Supplement: Supplementary file 1 [file insects-10-00245-s001.zip › Supplementary Materials/Table S7.docx]

**Table S7** Top 15 pathways enriched by *cis*-regulatory target genes of DElncRNAs in Am7CK vs Am7T.

| **Pathway** | **Number of enriched genes** |
| --- | --- |
| Longevity regulating pathway-multiple species | 3 |
| Pyrimidine metabolism | 3 |
| RNA polymerase | 2 |
| FoxO signaling pathway | 2 |
| Ribosome | 2 |
| Carbon metabolism | 2 |
| Ubiquitin mediated proteolysis | 2 |
| Protein processing in endoplasmic reticulum | 2 |
| Purine metabolism | 2 |
| Nitrogen metabolism | 1 |
| ABC transporters | 1 |
| Proteasome | 1 |
| DNA replication | 1 |
| Lysosome | 1 |
| Spliceosome | 1 |
