# Supplementary material for: Genome-Wide Identification of Long Non-Coding RNAs and Their Regulatory Networks Involved in Apis mellifera ligustica Response to Nosema ceranae Infection
Source: Insects. 2019 Aug 9;10(8):245. doi: 10.3390/insects10080245 (PMC6723323; doi:10.3390/insects10080245)
Supplement: Supplementary file 1 [file insects-10-00245-s001.zip › Supplementary Materials/Table S8.docx]

**Table S8** Top 15 pathways enriched by *cis*-regulatory target genes of DElncRNAs in Am10CK vs Am10T.

| **Pathway** | **Number of enriched genes** |
| --- | --- |
| Sphingolipid metabolism | 4 |
| Amino sugar and nucleotide sugar metabolism | 4 |
| mRNA surveillance pathway | 4 |
| Hippo signaling pathway-fly | 4 |
| Drug metabolism - other enzymes | 3 |
| RNA transport | 3 |
| Drug metabolism-cytochrome P450 | 2 |
| Endocytosis | 2 |
| Metabolism of xenobiotics by cytochrome P450 | 2 |
| Pentose and glucuronate interconversions | 2 |
| Fructose and mannose metabolism | 2 |
| Ubiquitin mediated proteolysis | 2 |
| Starch and sucrose metabolism | 2 |
| Hedgehog signaling pathway | 2 |
| Pyrimidine metabolism | 2 |
