# Supplementary figures and images for: Genome-Wide Identification of Long Non-Coding RNAs and Their Regulatory Networks Involved in Apis mellifera ligustica Response to Nosema ceranae Infection
Source: Insects. 2019 Aug 9;10(8):245. doi: 10.3390/insects10080245 (PMC6723323; doi:10.3390/insects10080245)

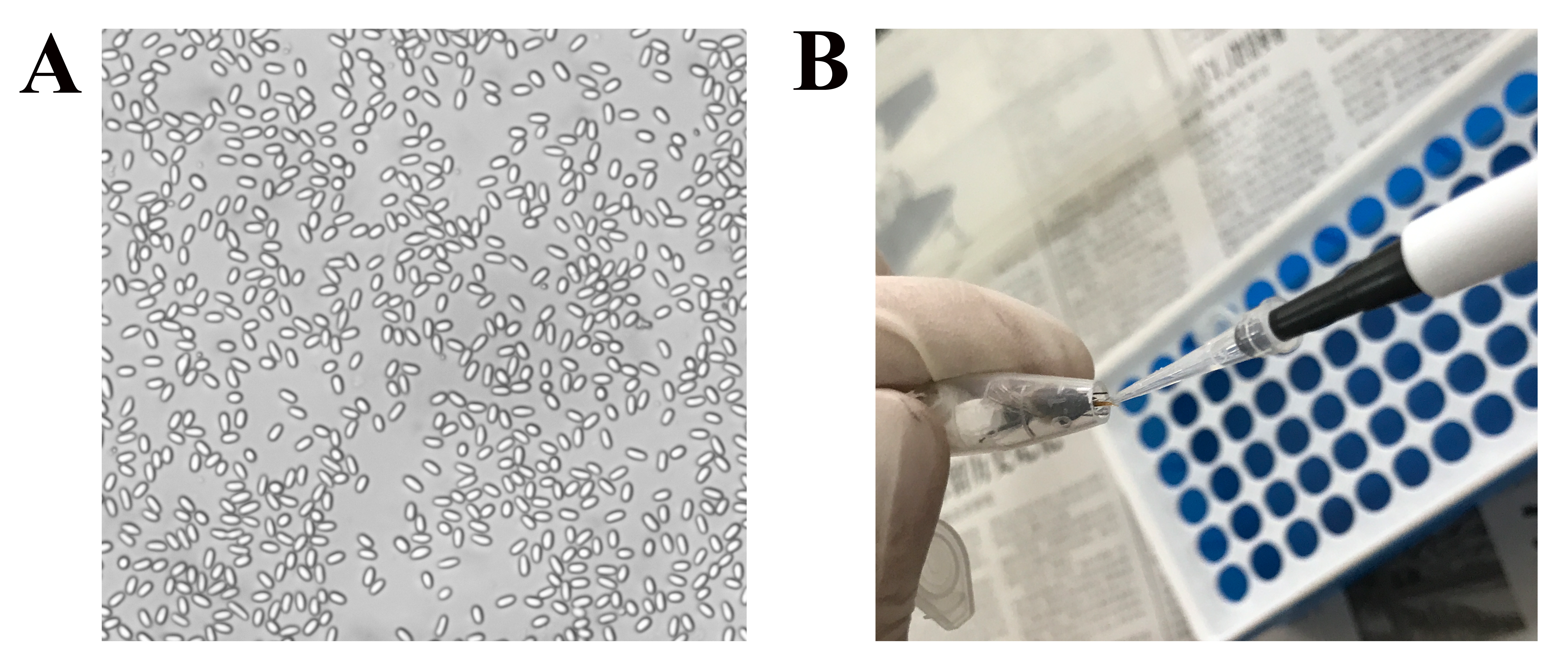

Supplement: Supplementary file 1 [file insects-10-00245-s001.zip › Supplementary Materials/Figure S1.tif]

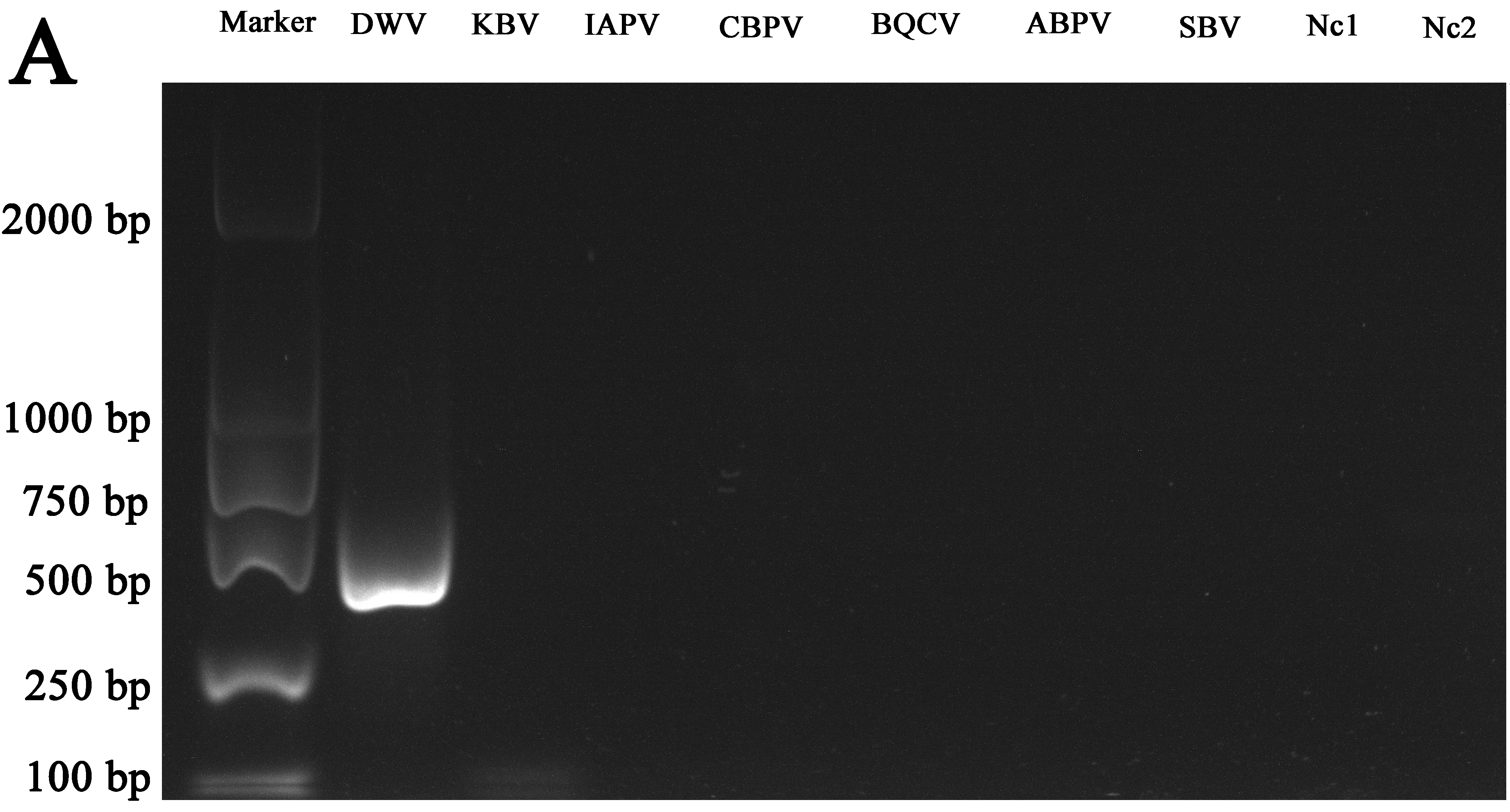

Supplement: Supplementary file 1 [file insects-10-00245-s001.zip › Supplementary Materials/Figure S2.tif]

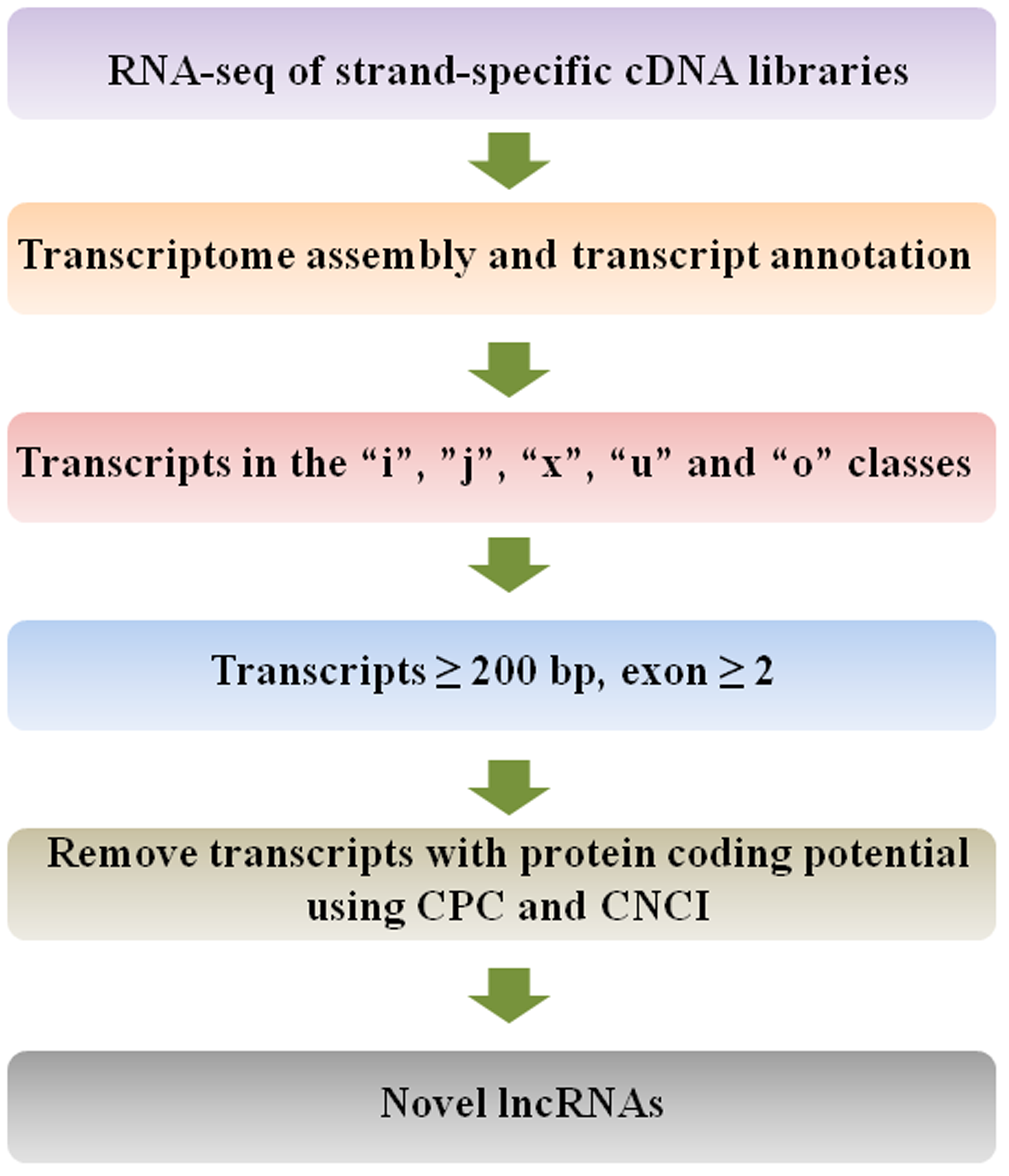

Supplement: Supplementary file 1 [file insects-10-00245-s001.zip › Supplementary Materials/Figure S3.tif]

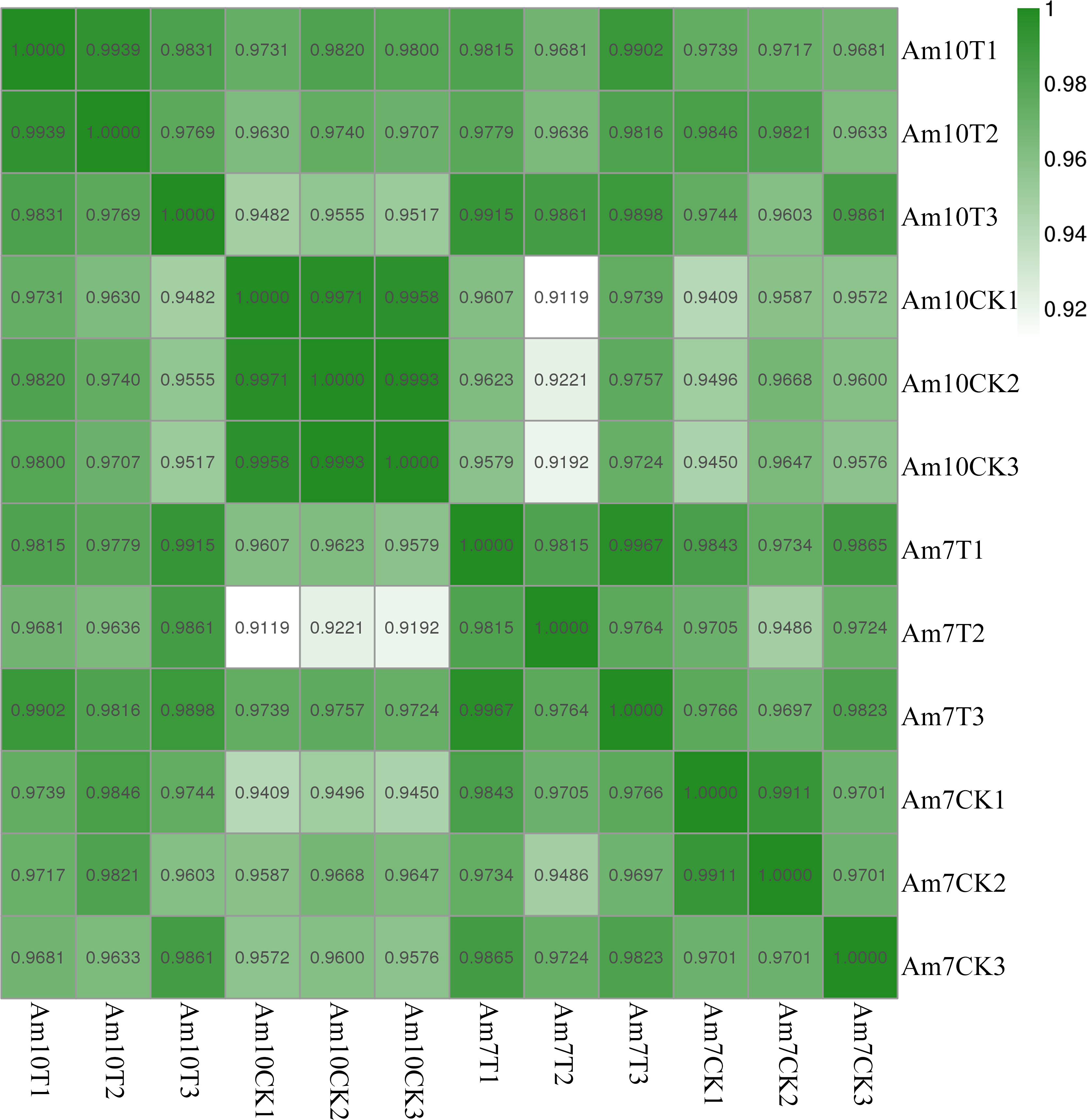

Supplement: Supplementary file 1 [file insects-10-00245-s001.zip › Supplementary Materials/Figure S4.tif]

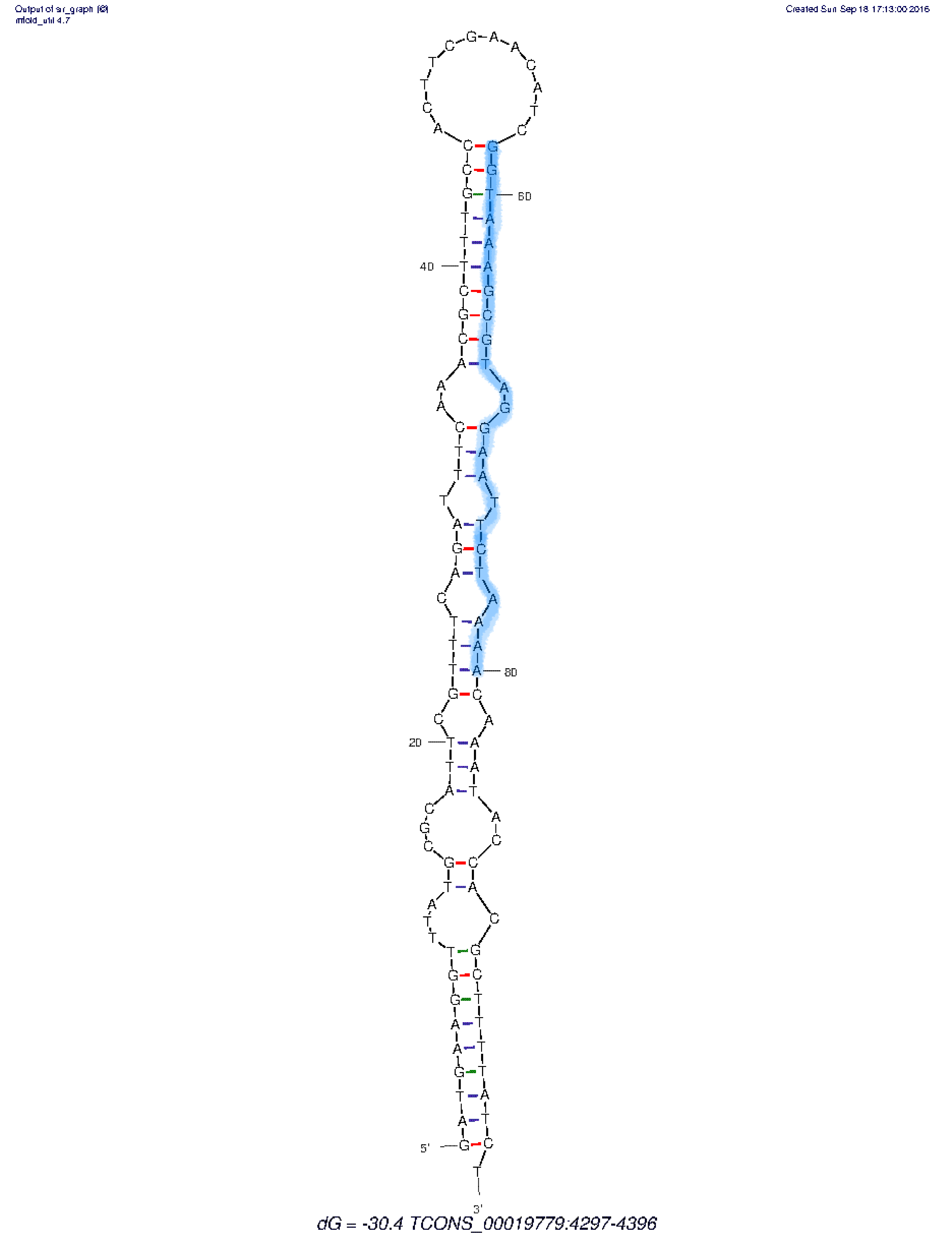

Supplement: Supplementary file 1 [file insects-10-00245-s001.zip › Supplementary Materials/Figure S5.tif]

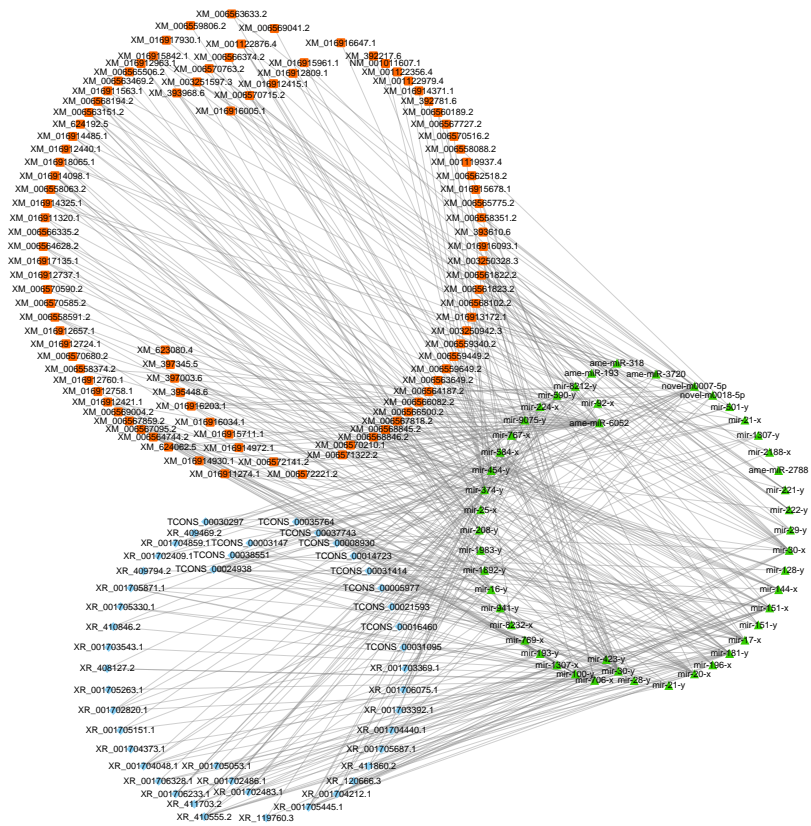

Supplement: Supplementary file 1 [file insects-10-00245-s001.zip › Supplementary Materials/Figure S6.pdf]

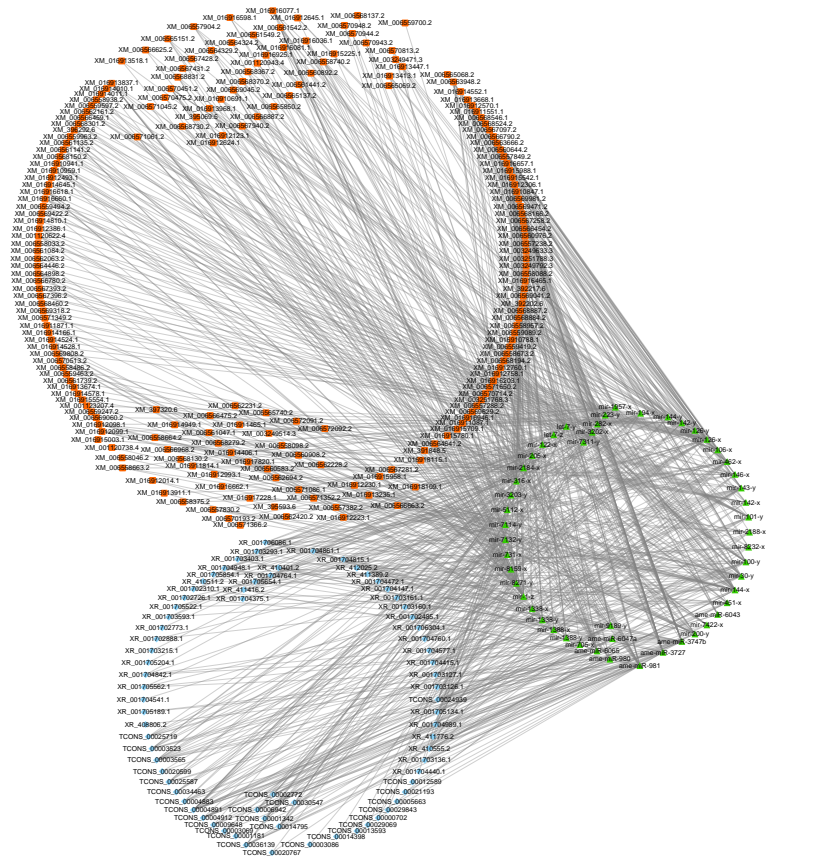

Supplement: Supplementary file 1 [file insects-10-00245-s001.zip › Supplementary Materials/Figure S7.pdf]
